# Bioaccumulation and Biomagnification of Mercury Along the Seafood Chain in Europe: A Systematic Review

**DOI:** 10.3390/foods14213752

**Published:** 2025-10-31

**Authors:** Riccardo Fioravanti, Luca Muzzioli, Eleonora Maurel, Giuseppe Palma, Giorgio Calabrese, Alberto Angioni, Cinzia La Rocca, Alberto Mantovani, Andrea Pezzana, Lorenzo Maria Donini

**Affiliations:** 1 Department of Experimental Medicine, Sapienza University, 00161 Rome, Italy; fioravanti.2029834@studenti.uniroma1.it (R.F.); luca.muzzioli@uniroma1.it (L.M.); 2 Clinical Epidemiology and Public Health Research Unit, Institute for Maternal and Child Health-IRCCS “Burlo Garofolo”, 34137 Trieste, Italy; 3 Assoittica Italia, 00161 Rome, Italy; 4 School of Medicine, Università del Piemonte Orientale, 28100 Novara, Italy; giorgiocalabrese@gcalabrese.it; 5 Italian National Food Safety Committee, Ministry of Health, 00153 Rome, Italy; aangioni@unica.it (A.A.); cinzia.larocca@iss.it (C.L.R.); alberto.mantovani1956@gmail.com (A.M.); andrea.pezzana@unito.it (A.P.); 6 Department of Life and Environmental Science, University of Cagliari, Campus of Monserrato, 09042 Cagliari, Italy; 7 Center for Gender-Specific Medicine, Istituto Superiore di Sanità, 00161 Rome, Italy; 8 Study Centre KOS-Science Art Society, 00144 Rome, Italy; 9 SC Nutrizione Clinica, ASL Città di Torino, 10128 Turin, Italy

**Keywords:** Hg contamination, methylmercury, bioaccumulation, environment, seafoods

## Abstract

Mercury (Hg) is a pervasive environmental contaminant with high bioavailability and toxicity, accumulating in aquatic food chains and posing significant risks to human health through seafood consumption. This systematic review aims to collect evidence on Hg bioaccumulation in seafood across Europe, assessing species that exceed legal limits. A total of 74 studies were identified on bioaccumulation among marine fish and seafood from European and adjacent seas, published between 2000 and 2024. Findings highlight that methylmercury (MeHg) constitutes the majority of total Hg in fish species, with concentrations often exceeding EU regulatory limits, especially in the Adriatic and Iberian areas. In general, teleosts exhibit higher tissue concentrations of both MeHg and total Hg compared to either selachians or mollusks. Species likely to exceed their legal limits are larger, apex predators, e.g., tuna, swordfish, and sharks, as well as benthic species, e.g., monkfish and mullet. In recent years, there has been a decrease in mercury contamination, probably due to agreed international regulations. However, significant regional variations still persist in Europe. To mitigate Hg contamination in seafood and ensure food safety, this study highlights the need for ongoing monitoring and management strategies, the interplay of environmental factors, food web dynamics, and species-specific biological characteristics.

## 1. Background

Mercury (Hg) is an ubiquitous environmental contaminant found in air, soil, water, and biological tissues. It exists in various chemical forms, including elemental mercury (Hg0), inorganic mercury (Hg salts), and organic mercury. The most prevalent form is methylmercury (MeHg) [1]. Mercury occurs in the environment due to natural sources such as volcanic activity and submarine tectonic activity, as well as anthropogenic activities, including industrial processes and waste disposal, such as dental amalgams. These have represented and still represent an important source of mercury in the environment [2]. Additionally, the European Environment Agency (EEA) has emphasized that climate change-driven events, such as permafrost melting, heavy rainfall, flooding, and forest fires, can release Hg-stored mercury in the environment, making it accessible to living organisms [3]. MeHg is a well-known food toxicant, of particular concern due to its ability to contaminate the seafood chains. It is produced through the bacterial methylation of inorganic Hg in aquatic sediments [1,4]. Aquatic organisms concentrate metals in their tissues from the water column and sediments, with accumulation rates influenced by the species’ ability to absorb and eliminate the element [5]. The high bioavailability of MeHg, stemming from its amphiphilic nature, enables efficient absorption and slow excretion in organisms. It exhibits a strong affinity for the thiol group (-SH) of the amino acid cysteine. By binding to cysteine, MeHg mimics methionine, entering the cellular systems where it exerts its toxic effects [1]. This mechanism enables storage across various tissues, including muscle [6]. Consequently, MeHg differs significantly from lipid-soluble contaminants such as dioxins and PCBs, which primarily bioaccumulate in adipose tissue. MeHg is efficiently transferred along aquatic food chains, resulting in elevated concentrations at higher trophic levels. Indeed, MeHg constitutes 75–100% of the total mercury (THg) present in the tissues of aquatic organisms [1].

Seafood, whether farmed or wild, is an important source of protein as well as of essential trace elements and vitamins, such as iodine and vitamin B12. In particular, it is a major source of omega-3 fatty acids, which are beneficial for brain development and cardiovascular health [7,8]. However, the intake of contaminants, particularly MeHg, may jeopardize the nutritional benefits of fish. According to EFSA, this depends on the level of contamination and the species of fish that is most commonly consumed [9,10]. Both marine and freshwater fish can be a source of MeHg; however, marine food chains are by far the main source of MeHg in the European diet [6]. Notably, MeHg is also a significant issue for farmed fish due to feed contamination; substituting animal-derived ingredients with vegetable ingredients has led to a substantial reduction in risk [11]. Therefore, ensuring adequate nutritional intake while limiting MeHg exposure is important for human health, making it a paradigm case for benefit-to-risk assessment [12]. In the European Union, compliance with the legal limits for Hg and other contaminants, as set out in European Regulation (EU) 915/2023, is monitored through official controls carried out in accordance with the Multi-Annual National Control Plans drawn up by each Member State [13]. The requirements for these plans are defined by Regulation (EU) 2017/625.

This systematic review aims to assess mercury (Hg) bioaccumulation in both wild-caught and farmed seafood across Europe through a comprehensive and up-to-date analysis of the scientific literature from 2000 to 2024.

## 2. Materials and Methods

The study was designed using the PECO model to investigate marine fish and seafood species, considering them as “Population”. The “Exposure” of these aquatic species to Hg and MeHg was defined as species exceeding or not exceeding the legal levels recommended by European Regulation (EU) 915/2023 on food contaminants acting as the “Comparator”. The “Outcome” was defined as the evaluation of Hg concentration in the food chain across European seas [13].

This systematic review protocol was registered with PROSPERO International Prospective Register of Systematic Reviews in September 2023 (protocol ID: 436929). The final data extraction took place in July 2024.

The study processes were guided by the Preferred Reporting Items for Systematic Reviews and Meta-Analyses (PRISMA) Statement [14].

The study search was conducted in PubMed, Web of Science, Scopus, EFSA, and Cochrane databases using the following search string:

(Hg OR “Total Hg”) AND (toxicity OR “food contamination” OR bioaccumulation OR biomagnification) AND Europe.

Inclusion criteria:
  - Studies conducted exclusively in Europe and neighboring countries whose food networks are closely linked to Europe for commercial and geographical reasons.
  - Studies conducted on marine fish products.
  - Studies conducted only on animals intended for human consumption.
Exclusion criteria:
  - All studies unrelated to Hg concentration.
  - All studies related to research on a single organism and/or living beings not intended for human consumption.
  - Studies proposing models of heavy metal accumulation.
  - Studies conducted on freshwater fish.
  - Studies conducted on European territories, but far from the continent itself.

The eligibility of the studies was assessed in two phases. Initial screening was performed using titles and abstracts, followed by a full-text review of potentially eligible studies. Any disagreements were resolved through discussion among the authors.

The following information was extracted for all studies and is included in Supplementary Table S1: first author, publication year; products; sample size; sampling location/sample origin; product’s part; total Hg min (mg/kg); total Hg max (mg/kg); total Hg mean (mg/kg); main result(s); and conclusions. To investigate the level of contamination in Mediterranean fish species, only studies on captured fish or fish purchased at markets were selected. An additional selection criterion was determining the Hg level in the edible parts (i.e., muscles) of the animal. Species exceeding the legal limits for Hg established by current EU regulations of 0.3–1.0 mg/Kg wet weight, depending on species (Regulation (EU) 2023/915 [13]), were identified and classified into four main categories based on Hg concentration level. In line with EFSA’s conservative approach, for bioaccumulation studies, when multiple samples were available for the same species and location or when samples differed only in sex or preservation method, the highest analytical value was selected for consideration.

The categories were as follows: (a) 0.4–0.59 mg/Kg of THg; (b) 0.60–1 mg/Kg of THg; (c) 1.01–1.49 mg/Kg of THg; (d) above 1.5 mg/Kg of THg (exceeding the legal limit). However, these groupings should be treated as indicative, as the Hg legal limits according to the regulation apply to wet weight (w.w.), whereas some of these studies measure THg on dry weight (d.w.).

Finally, to assess trends in mercury concentration among species compared to legal limits, the conventional vote-counting procedure was applied, dividing studies into two categories: those with results above the ML and those with results not exceeding the ML [15].

## 3. Results

**Figure 1:**
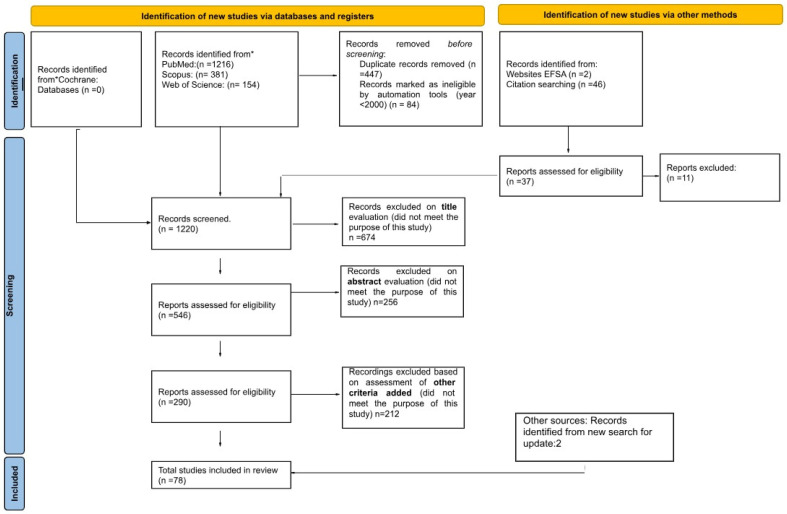
PRISMA flowchart reporting the study selection process. * Databases investigated for literature search with the string: (Hg OR “Total Hg”) AND (toxicity OR “food contamination” OR bioaccumulation OR biomagnification) AND Europe.

In July 2023 and July 2024, scientific literature was searched in the PubMed, Web of Science (WoS), Scopus, EFSA, and Cochrane databases. Meanwhile, the Cochrane databases yielded no results. The initial research on PubMed, Scopus, and WoS identified 1751 articles. After restricting the dataset to studies published from the year 2000 onwards and removing duplicates, 1220 articles were screened. An additional 37 relevant records were identified in the Journal of the EFSA and its citations. After screening the title and abstract, 674 articles were excluded, leaving 290 studies. Applying exclusion criteria based on species type (non-marine fish) led to the removal of an additional 212 studies. The final dataset comprised 78 studies focused on bioaccumulation (Figure 1).

Initially, the studies were categorized by the location at which seafood products were captured and/or sampled, including specimens sold at fish markets. Sixty studies were conducted in the Mediterranean area, while 22 were conducted in non-Mediterranean areas (the Atlantic and Arctic Oceans, the North Sea, the Baltic Sea, and the Black Sea). The variety of species investigated, the number of sampling locations, and the lack of reporting of sample sizes in most studies investigating Hg bioaccumulation in non-Mediterranean areas made it difficult to conduct a meta-analysis. Nevertheless, the dataset could be useful for food safety investigations, as it provides an overview of Hg content in seafood marketed in Europe and consumed by European consumers.

The findings indicate an overall decline in exceedances of legal limits with respect to geographic distribution and time trend. Between 2000 and 2012, 33.33% of analyzed samples recorded Hg levels exceeding legal limits. In the following years, up to 2024, this percentage of exceedance decreased, stabilizing at 28% (Table 1).

Meanwhile, significant regional variations persist across Europe, particularly around the Adriatic, the Ionian, and the Balearic Seas. Of the 84 species considered by the included studies, only eight showed an Hg level that exceeded ML. Notably, species with the highest residue values in category d) (≥1.5 mg/Kg) are the large predators *Thunnus thynnus* (tuna), *Xiphias gladius* (swordfish), and *Merluccius merluccius* (haddock), as well as the omnivorous benthic *Mullus barbatus* (mullet). The highest recorded value (3.37 mg/Kg) was found in *Thunnus thynnus* (Table 2).

The results of the vote-counting procedure indicate that a majority of studies on *Conger conger*, *Lophius* genus, and *Thunnus alalunga* showed median values exceeding the maximum legal limit (ML). On the contrary, *Mullus barbatus* and *Xiphias gladius* reported study percentage and median values under ML. Lastly, *Thunnus thynnus* and *Merluccius merluccius* showed conflicting results (Table 3).

### 3.1. Mediterranean Area Contamination

Approximately 30% of studies (18 studies out of 60) concerning the Mediterranean area reported Hg levels exceeding the legal limits [20,23,32,33,34,35,36,37,38,39,40,41,42,43,44,45,46,47,48,49,50,51,52,53,54,55]. Most of these studies focused on the Adriatic region. Fifty-two different species of fish exceeded the legal limits. Some benthic predators were found to be highly contaminated, with specimens of the *Scorpaena* genus exhibiting exceptionally high levels of Hg (up to 4.40 mg/Kg d.w.) [21,36] and species from the *Lophius* genus displaying contamination values ranging from 0.68 mg/Kg to 1.26 mg/Kg (*n* = 615) [16]. Regarding apex demersal predators, the *Thunnus* genus had 632 samples that exceeded the limits, showing Hg levels ranging from 1.02 mg/Kg to 3.37 mg/Kg [4,23]. *Xiphias gladius* exhibited Hg levels above the legal limit, ranging from 1.04 mg/Kg to 2.41 mg/Kg (*n* = 34) [25,56].

A study by Di Leo et al. found that Hg levels are significantly lower in mollusks than in fish [56]. Similarly, the THg/MeHg ratio is lower in mussels than in fish (from 20% to 60%), whereas in fish it ranges from 80% to 100% [57]. In some cases, bivalve mollusks and cephalopods exceeded the limit permitted by current EU regulations [38,58]. However, bivalve mollusk samples generally contain less Hg than the legal limit [59,60] (Table S1).

### 3.2. Non-Mediterranean Area Contamination

In the Baltic Sea, even species that are not strictly native exhibit a pronounced tendency to bioaccumulate and biomagnify THg, and particularly MeHg, along the marine food web. Concentrations increase with organismal age and size (length), as well as with trophic level, with apex predators displaying the highest Hg burdens [61,62,63]. The species analyzed include flatfish such as *Scophthalmus maximus* and crustaceans such as *Rhithropanopeus harrisii*. Although they demonstrate an excellent ability to bioaccumulate MeHg, they never exceed the legal limits for Hg [61,64].

In the Atlantic Ocean, shortfin mako sharks (*Isurus oxyrinchus*) and blue sharks (*Prionace glauca*) caught in the northeast Atlantic have been found to have significantly higher Hg concentrations, reaching 2.57 mg/Kg and 1.71 mg/Kg, respectively [65,66]. Detailed assessments indicate that the majority of mako sharks exceeding 190 cm in length and blue sharks exceeding 250 cm surpass the regulatory threshold of 1 mg/Kg established by the European Union. Previous reviews have reported that oceanic apex predator fish species have the highest Hg concentrations, with greenish (*Prionace glauca*) averaging 0.97 mg/Kg and species of the genus *Thunnus*, such as *Thunnus thynnus* (0.71 mg/Kg) and *Xiphias gladius* (0.57 mg/Kg), being particularly affected [1,67].

Similarly, swordfish (*Xiphias gladius*) have been found to contain Hg concentrations of up to 1.74 mg/Kg. Overall, 22% of examined shark and swordfish samples (*n* = 37) exceeded the 1 mg/Kg Hg limit [27]. Other commercially important species, such as tuna and conger eel (*Conger conger*), exhibited considerable Hg concentrations, albeit with variability, thus contributing to human exposure through diet. MeHg was the predominant chemical species, constituting an average of 88.1% of total Hg in the considered species [61,68,69,70,71] (Table S1).

### 3.3. Farmed Fish

A significant amount of the fish consumed in Europe is farmed. This review did not specifically consider farmed fish. However, a recent systematic review highlighted that farmed species typically exhibit lower Hg concentrations than their wild counterparts [72,73]. Only four of the selected studies considered farmed *Thunnus thynnus* (Atlantic bluefin tuna) specimens. Three of these studies found that none of the samples surpassed the EU regulatory limit [74,75,76], whereas the study by Milatou et al., conducted in Greece, revealed that 40% of muscle tissue samples from farmed specimens contained Hg levels exceeding the European Commission’s maximum allowable limit of 1 mg/Kg [77]. Approximately 38% of *Thunnus* samples showed Hg concentrations above legal thresholds (ranging from 1.02 mg/Kg to 1.89 mg/Kg) [77]. Similarly, none of the samples from the studies on farmed *Merluccius merluccius* surpassed the legal limit [74,75,76].

## 4. Discussion

Based on the selected studies, Hg contamination of fish in European marine areas is a significant issue, with levels varying depending on species, geographic location, and environmental factors. Using the EU legal limits as a pragmatic benchmark highlights the species and ecological niches that are more susceptible to Hg bioaccumulation.

Of the included species, eight demonstrated values exceeding the ML, and only four showed consistent exceedances across studies. Teleost fish tend to have higher levels of both MeHg and total Hg than other taxa. Data indicate that in the European region, species with higher tissue levels belong to two clusters: large predators (e.g., tuna and swordfish) and benthic species, including both predators (e.g., monkfish) and omnivores (e.g., mullet). Biomagnification along the marine food chain appears as a critical factor associated with higher tissue bioaccumulation [74,78]. The benthic ecosystem, which is associated with prolonged contact with sediments, is also important. Cartilaginous fishes, such as Lamnidae/sharks, are top open-sea predators and bioaccumulate Hg, albeit at consistently lower levels compared to teleosts. The same conclusion applies to mollusks, which can be either carnivorous (e.g., cephalopods) or bottom-dwelling filtering organisms (e.g., lamellibranches) [79]. Mollusks also consistently exhibit a lower rate of MeHg to total Hg ratio than vertebrates. Overall, alongside the ecological niche, differential Hg bioaccumulation appears to be associated with differences among zoological taxa.

Previous reviews have highlighted concerns about Hg pollution in seafood and marine ecosystems. This has led to a growing commitment to assessing the most contaminated species, the amount of Hg in food products, and its impact on human populations. Consequently, numerous studies have identified the primary factors that significantly impact Hg contamination in fish fauna, including size, age, weight, trophic position, and food chain length. By contrast, factors such as sex and the proportion of MeHg to Hg in sediment have shown weaker correlations with Hg accumulation in fish [1,80,81].

This review, which is based on studies focusing on European seas and published between 2000 and 2024, highlights the importance of benthic ecosystems as an additional key factor. Indeed, the methylation of Hg to form MeHg mostly takes place in water sediments [6]. Values found in pelagic species are 2–4 times higher than those reported in previous publications [16,17], where most common seafood products fall within the limits set by the EC. For example, *Merluccius merluccius* was found to bioaccumulate Hg beyond the permitted limits in several studies conducted in the Adriatic Sea, the Almeria region of Spain, and the Gulf of Lion [27,29,30,31]. Additionally, the results obtained for imported fresh fish exceed the legal limits [82]. Therefore, these findings indicate the ongoing need for attention to be given to Hg pollution in Europe [83].

Farmed species typically show Hg concentrations below those detected in wild counterparts [72,73]. The exposure of farmed fish mainly depends on their feed, the composition of which is regulated by specific legal limits on metal content. Therefore, innovation in aquaculture feed ingredients has led to a reduction in contaminant levels [11]. Conversely, the levels of Hg in caught fish reflect the environmental quality of European water bodies. This is due not only to bioconcentration but also to biomagnification, and thus caught fish represent a major link between Hg emissions and humans in ecosystems, as well as being a factor in the safety of the human food chain [6]. The peculiarities of long-lived, high-trophic-level marine predators may also reflect bioaccumulation and biomagnification processes. For example, Milatou et al.’s study found that 40% of analyzed species exceeded safety limits. However, this could be due to a methodological bias: rather than analyzing all individual samples separately, researchers opted to analyze pooled samples by aggregating size and farming period to lower analysis costs [77,82].

Two species of the genus *Lophius* have shown a significant exceedance of ML: *Lophius piscatorius* and *Lophius budegassa*. For these species caught in the Adriatic Sea, it is noted that the levels of MeHg are particularly high and consistent across the analyzed studies [16,17,18,19]. This review revealed a positive correlation between weight, length, and the presence of Hg, particularly in benthic ecosystems and demersal fish [47,84,85]. These species tend to accumulate more Hg compared to pelagic fish due to their feeding habits and their position in the trophic chain [18,86]. In addition, alongside the trophic level, the physiology and metabolism of marine species can influence Hg distribution across different tissues, due to proteins rich in specific amino acid groups that can bind different Hg forms [57].

Regarding geographic distribution, this review clearly shows that regional variations persist in Europe, suggesting that geochemical or anthropogenic emissions may still contribute to local or regional contamination hotspots. For instance, many of the samples that exceed legal limits originate from the Adriatic Sea. This phenomenon may be the result of a combination of factors, including the presence of cinnabar deposits in the Adriatic Sea and historical pollution from intense mining activities, such as those in Idrija that affect the Gulf of Trieste, and species-specific biological factors that favor the accumulation of this toxic element. However, this result may reflect research density rather than pollution severity [21,24,74].

Although all edible aquatic species are susceptible to MeHg deposition in their tissues, significant differences are driven by biology. Aquatic animals absorb Hg through both branchial respiration and dietary intake. Larger marine predators tend to exhibit the highest concentrations of Hg due to their higher trophic level, increased food consumption, requirement for animal proteins, and longer lifespans. Indeed, longer-living animals tend to have higher concentrations of MeHg [78]. Consequently, frequently consumed large predatory species such as *Thunnus thynnus* and *Xiphias gladius* (swordfish) are of particular concern. The EFSA notes that such species are high in MeHg and relatively low in omega-3, so high consumption could outweigh the benefits [10]. Studies also suggest that demersal fish tend to accumulate higher levels of metals than other types of fish [18]. Arctic populations have a diet rich in fish and marine mammals, so these individuals are highly likely to be exposed to Hg, which has an impact on their health [6].

Between 2000 and 2012, 26.92% of analyzed samples recorded Hg levels exceeding legal limits. In the following years, up to 2024, this percentage decreased, stabilizing at 28.5%. During the time frame under consideration, the approval of well-defined policy choices occurred, such as the Minamata Agreement in 2013 and the EU Decision 2017/939 of the European Council [87]. However, this review found no direct evidence of the influence of these policies and the related regulatory measures on the decrease in Hg concentration observed in the results. These measures include banning the opening of new Hg mines and restricting existing ones, gradually phasing out Hg-containing products, and limiting their use in manufacturing processes [87]. Strengthened controls have also been implemented on atmospheric emissions and water and land discharges. Additionally, the issue of artisanal gold mining using Hg has been addressed by promoting protocols for the safe storage and disposal of Hg-containing waste.

However, Hg accumulation does not occur uniformly across all fish species. Consequently, it is important to consider not only the environmental factors influencing Hg accumulation but also species-specific features. When considering significant factors in Hg bioaccumulation, it is crucial to account for anthropogenic Hg emissions, food web dynamics, and trophic shifts in deep-sea marine fish species simultaneously [83]. While some studies have found no correlation between age, weight of the samples, and the presence of Hg in their muscle tissue [61,81,88,89], there is a substantial body of literature reporting a statistically significant correlation [4,22,47,64,84,90]. Another factor influencing the presence of Hg in fish is the level of pollution at the site where the animals live. There is a statistically significant correlation between pollution at the fishing site and the presence of this contaminant in fish [91]. Bivalve mollusks are benthic and sessile organisms for most of their life cycle and are therefore directly exposed to contaminated sediments. These organisms primarily become contaminated through the filtration of water and the absorption of contaminants present in suspended particulate matter that settles on sediments [58]. However, the degree of contamination varies significantly depending on the specific contaminant: while Hg levels are generally low, bivalves exhibit a considerable capacity to accumulate lead and cadmium [47].

Hg is present in high concentrations in organs such as the liver and kidneys, which are responsible for eliminating toxicants in animals, and its concentration is influenced by the organism’s ability to metabolize and eliminate it [77]. However, muscle contamination is not directly correlated with contamination of these organs [24]. Furthermore, it was not possible to assess the risk of exposure to, or simply the presence of, Hg in marine fauna by aggregating the data by region, due to the extreme heterogeneity of the studies. Nevertheless, many studies report exceedances of safety [56,92,93,94,95,96,97,98,99] and the legal limits [19,29]. This is particularly evident around the Iberian Peninsula, from the Mediterranean and the Atlantic sides, as well as the Adriatic area.

Despite Hg, particularly MeHg, remaining a concern for food safety in Europe, regulatory measures and the Minamata Convention have been implemented. Nevertheless, large predatory species such as swordfish (*Xiphias gladius*) and tuna (*Thunnus* spp.) and benthic species such as monkfish (*Lophius* spp.) and mullet still exhibit significant levels of Hg, often surpassing the thresholds established by the European Commission. It is therefore essential to maintain and strengthen monitoring programs and periodically reassess the maximum legal limits for Hg in foods. Future strategies must adopt a holistic approach, balancing the potential risks of MeHg contamination with the significant nutritional benefits of fish consumption, such as proteins, iodine, selenium, and omega-3. In fact, despite several instances of legal limits being exceeded, it is not possible to assert that fish consumption needs to be reduced without further robust studies assessing the risk. This is because substantial literature recognizes that regular consumption of fish and fish products provides numerous benefits [9]. Therefore, it is essential to maintain and strengthen monitoring programs and periodically reassess the maximum legal limits for Hg in foodstuffs to reduce Hg exposure whilst maintaining the benefits of fish intake.

Limitations of the Study:
  - The vast majority of the selected studies were retrospective in design. This approach may introduce potential biases and limit the ability to establish causal relationships.
  - The lack of prospective studies in the selection may restrict the ability to predict future trends or outcomes accurately.
  - There was significant variability among the included studies in terms of methodologies, sample sizes, and reporting standards. This heterogeneity makes it challenging to draw consistent conclusions across the entire body of research.
  - The initial analysis revealed considerable difficulties in comparing analytical outcomes due to temporal and geographical differences between studies. These disparities may affect the generalizability of the findings and complicate the interpretation of trends over time or across regions.
  - Data presentation bias: Some of the included studies exhibited bias in the presentation of data, such as selective aggregation or reporting of results, which may have affected the clarity and interpretability of the findings.

## 5. Conclusions

The findings of this systematic review reveal that Hg contamination remains a cause of concern in European seas, as indicated by levels exceeding EU legal limits in some common edible species, thus posing a potential risk to consumers. Hg bioaccumulates in all marine organisms, with teleosts being more susceptible than other taxa. Among teleosts, open-sea predators and some benthic species consistently exhibit higher levels. Continued monitoring of at-risk teleost species, such as tuna, swordfish, haddock, and monkfish, should support ongoing vigilance and targeted studies. Further investigations into Hg in seafood must follow harmonized protocols regarding sampling, analysis, and reporting. This research also emphasizes that effective management strategies for Hg contamination in seafood must consider the complex interplay of environmental factors, food web dynamics, and species-specific biological characteristics that drive bioaccumulation. The authors of this review agree with the majority of the analyzed studies that recommended maintaining and implementing monitoring programs for this food contaminant. It would also be advisable to re-evaluate the maximum legal limits allowed in seafood.

## Figures and Tables

**Table 1:** Hg-contaminated samples between 2000–2011 and 2012–2024, divided per level of exceedance in respect to EU legal limits.

Level of Exceedance in Respect to EU Legal Limits:	n. of Samples (%)	
mg/Kg w.w.	2000–2011	2012–2024
(a) 0.0–0.59	21 (26.58%)	47 (20.61%)
(b) 0.60–1.00	1 (1.27%)	3 (1.32%)
(c) 1.01–1.49	2 (2.53%)	5 (2.19%)
(d) ≥1.50	3 (3.80%)	10 (4.39%)
Samples exceeding EU legal limits	27 (34.18%)	65 (28.51%)
Overall analyzed samples	79	228

**Table 2:** Summary of Hg contamination in selected fish species, with consistent exceedances of the EU regulatory limits.

Reference (Year)	Sampling Location	Species Analyzed	Sample Size	Range of Maximum THg Level (mg/Kg ww) Detected in Studies
Storelli (2000) [16]	Adriatic Sea	*Lophius budegassa* ^1^ ^(Monkfish)^	461	0.74–1.26
Storelli (2003) [17]	Adriatic Sea			
Storelli (2013) [18]	Adriatic Sea			
Storelli (2000) [16]	Adriatic Sea	*Lophius piscatorius* ^1^ ^(Monkfish)^	154	0.76–1.26
Llull (2017) [19]	Balearic Sea			
Storelli (2002) [4]	Adriatic and Ionian Seas	*Thunnus thynnus* ^2^ ^(Tuna fish)^	368	1.02–3.37
Licata (2004) [20]	Tyrrhenian and Ionian Seas			
Di Lena (2017) [21]	Adriatic and Tyrrhenian Seas			
Annibaldi (2019) [22]	Mediterranean Sea			
Kljaković-Gaspić (2021) [23]	Adriatic Sea			
Storelli (2004) [24]	Adriatic Sea	*Thunnus alalunga* ^2^ ^(Tuna fish)^	264	1.17–1.56
Storelli (2002) [4]	Adriatic and Ionian Seas			
Damiano (2011) [25]	Northwestern and North-central Atlantic, Tyrrhenian and Ionian Seas	*Xiphias gladius* ^2^ ^(Swordfish)^	34	1.04–2.41
Storelli (2013) [18]	Adriatic Sea	*Conger conger* ^1^ ^(Conger)^	180	0.56–1.14
Llull (2017) [19]	Balearic Sea			
Bonsignore (2013) [26]	Ionian Sea	*Mullus barbatus* ^2^ ^(Mullet)^	142	1.11–1.91
Harmelin-Vivien (2009) [27]	Gulf of Lion and Romanian Black Sea			
Di Bella (2020) [28]	Ionian Sea			
Sánchez-Muros (2018) [29]	Balearic Sea	*Merluccius merluccius* ^1^ ^(Haddock)^	456	0.59–1.67
Cossa (2012) [30]	Northeastern Atlantic and Gulf of Lion			
Perugini (2009) [31]	Adriatic Sea			

^1^ Legal limit 0.5 mg/Kg Commission Regulation (EU) 2023/915; ^2^ Legal limit 1.0 mg/Kg Commission Regulation (EU) 2023/915 [13]. Abbreviations: THg: total mercury; ww: wet weight.

**Table 3:** Summary of Hg contamination in fish species exceeding ML, including median values, studies with positive results, and percentage of samples above limits.

Species	No. of Studies	Overall Sample Size (N)	Median (IQR)	Studies with Positive Result (%)	No. of Samples Above ML (%)
*Conger conger* ^1^ ^(Conger)^	2	180	1.14 (1.14–1.14)	2 (100%)	180 (100%)
*Lophius budegassa* ^1^ ^(Monkfish)^	3	461	0.76 (0.68–0.76)	3 (100%)	461 (100%)
*Lophius piscatorius* ^1^ ^(Monkfish)^	3	160	1.26 (1.00–1.26)	3 (100%)	154 (96.3%)
*Merluccius merluccius* ^1^ ^(Haddock)^	8	602	1.67 (0.59–1.67)	3 (37.5%)	461 (76.6%)
*Mullus barbatus* ^2^ ^(Mullet)^	10	1271	0.31 (0.43–0.70)	2 (20%)	138 (10.9%)
*Thunnus alalunga* ^2^ ^(Tuna fish)^	3	346	1.17 (1.17–1.56)	2 (66,7%)	264 (76.3%)
*Thunnus thynnus* ^2^ ^(Tuna fish)^	9	982	0.87 (0.86–1.02)	5 (55.6%)	384 (39.1%)
*Xiphias gladius* ^2^ ^(Swordfish)^	5	292	0.49 (0.49–0.62)	2 (40%)	35 (12%)

^1^ ML = 0.5 mg/Kg ww; ^2^ ML = 1.0 mg/Kg ww. Abbreviations: IQR: Inter Quartile Range; ML: Maximum Limit; ww: wet weight.

## Data Availability

The original contributions presented in this study are included in the article/Supplementary Materials. Further inquiries can be directed to the corresponding author.

## Supplementary Materials

The following supporting information can be downloaded at https://www.mdpi.com/article/10.3390/foods14213752/s1, Table S1: Data extraction table. All references in Supplementary Material have been cited in the main text.
