# Supplementary material for: Bioaccumulation and Biomagnification of Mercury Along the Seafood Chain in Europe: A Systematic Review
Source: Foods. 2025 Oct 31;14(21):3752. doi: 10.3390/foods14213752 (PMC12610611; doi:10.3390/foods14213752)
Supplement: Supplementary file 1 [file foods-14-03752-s001.zip › foods-3921825-supplementary.pdf]

Table S1. Data extraction table

First author, year [cit]	Product/s	Sample size	Sampling location/sample origin	Product's part	Total Hg Min (mg/kg)	Total Hg Max (mg/kg)	Total Hg Mean (mg/kg)	Main result(s)	Conclusions
**Acquavita A. 2018 [89]**	*Zosterisessor ophiocephalus*	208	The Marano and Grado Lagoon (northern Adriatic Sea, Italy)		0.48	1.67	0.61	- No significant direct correlation total Hg content with size expressed in terms of Ltot taking into con sideration the entire population a significant correlation was found for females. This can be explained by taking into consideration the ethology of this species: in spring males defend the nest, while females are very active and increase their exploring territory This is evident especially in spring, which is the reproductive season. It can be hypothesised that the need for the energy necessary for egg production. The authors also observed that the females of this species exhibit higher Hg concentrations than the males.	- Hg is widely diffused in the Marano and Grado Lagoon. Z. ophiocephalus exceed the legislated standards for human consumption.
**Ancora S. 2020 [40]**	*Tetrapturus belone*	29	Tyrrhenian Sea, Strait of Messina and Ionian Sea	muscle	0.786	8.56	3.40	- Significant positive correlations between element concentrations and sizeHg in liver (rs = 0.713, p < 0.01) and muscle (rs = 0.452, p < 0.05)<br>- Significant correlation between muscle/liver ratios for Hg and Se (rs = 0.458, p < 0.05).	- Trophodynamic studies using stable isotopes may provide further knowledge on the ecology of this species and on the contribution of different prey species in Hg and other trace element accumulation.
**Annibaldi A. 2019 [22]**	*Thunnus thynnus L. farmed*	40	Mediterranean Sea - Malta	Muscle			0.61	- Farmed tuna showed Hg levels below the legal limit (0.6 ± 0.2 mg/kg) and higher Se concentrations (1.1 ± 0.9 mg/kg).<br>- Wild tuna had Hg levels exceeding the legal limit (1.7 ± 0.6 mg/kg) and lower Se (0.6 ± 0.3 mg/kg).<br>significant positive correlation between Hg and weight was observed only in wild tuna	- Farmed Atlantic bluefin tuna has a minor risk/benefit ratio and is safer than wild tuna.
	Thunnus thynnus L.) wild	33	Mediterranean Sea - Sardinia	muscl e			1.7		
**Azad AM. 2019 (60)**	*Molva dypterygia*	41	The Hardangerfjord fjord in Norway				1.44	- Detailed results indicate that tusk and blue ling from the inner Hardangerfjord showed the highest mean Hg concentrations (1.87 and 1.44 mg kg−1 ww), with all individual samples exceeding the EUML.<br>- Sediments near the point source (Sørfjord) were highly contaminated, tusk in the adjacent Eidfjord branch exhibited similarly high Hg levels, suggesting that not only the point source but also terrestrial run-off, atmospheric deposition, and hydroelectric power stations are important sources of contamination for biota.	- Concentrations were ten times higher in the inner fjord branch with a PSP (Sørfjord) compared to an adjacent fjord branch that may have been influenced by freshwater inputs
	Brosme brosme	138					1.08		
	Molva molva	30					1.89		
	Anarhichas lupus	4					0.03		
	Sprattus sprattus	5					NR		
	Cancer pagurus	20					0.22		
	Homarus gammarus	26					0.62		
	Nephrops norvegicus	10							
**Besada V. 2010 (70)**	*Mytilus galloprovincialis*	2050	Spain				0.18	-Across 41 sampling sites reported (50 mussesl for sample) highly similar patterns in the NW and N Spanish regions.<br>-Northern area showed higher median concentrations for Hg and Pb than the Atlantic areas, correlating with the presence of present or former mining activities and/or large industrial facilities.	- Decreasing trend in the concentrations of several metals (mainly, Hg), which were strongly related to anthropogenic activities (mining and road traffic<br>-Strict regulations and environmental protection activities enforced during the last years are having a positive effect on reducing
**Biton-Porsmoguer S. 2018 (65)**	*Prionace glauca*	40	north-eastern Atlantic (Vigo Market)		0.14	1.71	0.52	- Hg concentration increased significantly with size and weight in both species, as significant linear relationships were observed between log Hg and TL, (cm) and between log Hg and total weight in blue shark; in shortfin mako.<br>- Diet is recognized as the main pathway of Hg intake in high-level predators	- Hg content above this legal threshold was recorded in the majority of adult blue shark larger than 250 cm TL and shortfin mako larger than 190 cm TL.<br>- The suggestion is to avoid the capture and commercialization of individuals exceeding these respective lengths for the two species.
	Isurus oxyrinchus	48			0.12	2.57	0.74		

**Biton-Porsmoguer S. 2022 (49)**	*Xiphias gladius*	26	Catalan Sea (north-western Mediterranean Sea	Stomachs, gonads and livers				- The poor condition of swordfish based on size and the levels of lipid in the liver. and the high Hg levels accumulated to the trophic web (particularly from cephalopods) may indicate potential unfavourable feeding and reproduction conditions for swordfish in the NW Mediterranean and this warrants further investigation.	- The swordfish condition (in terms of liver size and liver lipid content) seems to be impaired in the Catalan Sea compared to other areas of the Mediterranean Sea, indicating a potentially unfavorable food supply that should be explored in future studies.<br>- Cephalopods, main prey of swordfish, in summer in the Catalan Sea and the main vector of Hg accumulation in swordfish. The mean mercury levels of swordfish remain below the EU maximum regulatory levels.
**Blanco SL. 2008 (61)**	*Thunnus alalunga canned*	75	Galicia, Spain	Muscle	-	1.2	0.21	- These results demonstrate that P. maximus has actually a strong capacity to bioaccumulate Hg from environment.	- The level of heavy metals in seafood samples from various Spanish processors and distributors has shown that, marketed products are safe according to European legislation.<br>- The high levels of mercury in some species, such as swordfish and sharks, and the relatively important contribution to mercury ingestion of widely consumed species, such as tuna, need to be considered when dealing with particularly sensitive sectors of the population.<br>- The Hg concentrations in edible tissues (adductor muscle and gonad) were much lower than the safety limits
	Thunnus alalunga frozen	15			0.12	0.75	0.18		
	Thunnus Albacares canned	109			-	0.87	0.24		
	Thunnus Albacares frozen	21			-	1.02	0.4		
	Thunnus thynnus canned	113			-	0.87	0.17		
	Thunnus thynnus frozen	45			-	0.49	0.27		
	Prionace glauca	13			0.6	1.4	0.97		
	"Marlin"	2			0.81	0.95	0.88		
	Xiphias gladius	24			-	1.74	0.68		
	Sardina pilchardus	31			-	-	-		
	Scomber scombrus	15			-	0.12	-		
	Engraulis encrasicolus	9			-	0.18	-		
	cephalopod mollusks	21			-	0.1	-		
	bivalve mollusks	34			-	-	-		
	Pecten maximus	40	Normandy, French North Atlantic coast						
**Bonsignore M. 2013 (26)**	*23 Differtent species*	21	Augusta bay (sicily)	muscle liver				- Founded high total mercury concentrations in benthic fish from Augusta Bay, suggesting an active release of mercury from polluted sediments into the water column and subsequent bioaccumulation within the food web.<br>-Pelagic species in the external bay showed high contamination, confirming Augusta Bay as a significant source of Hg for the surrounding marine environment and highlighting the risk of contaminant transfer to the open sea.	- The findings also suggest caution in consuming demersal and benthic fish from outside the bay, underlining the need for appropriate social actions due to the contamination.<br>- HgT levels in fish increased with habitat depth, with benthic species showing the highest values.<br>- Liver HgT concentrations were also 1.5 to 6 times higher than in muscle
**Boquete MT. 2023 (86)**								- FoundHg biomagnification across trophic levels, even when Hg concentrations in the prey were relatively low.<br>- The calculated trophic magnification factors (TMFs) for total Hg were notably high, comparable to those typically found for methylmercury.<br>- The fraction of methylmercury (MeHg) relative to total Hg likely increases under higher Hg contamination levels.	- Provided robust evidence of Hg bio magnification even when this element was found at relatively low concentrations in the prey.

**Burioli EAV. 2017 (91)**	*Crassostrea gigas*	30	Muggia gulf		0.12	0.14	0.13	- All the samples, the concentration of the elements, Cd, Hg and Pb, was below the maximum limit for food set by European Regulations.<br>- The Hg concentration was below the detection limit in the samples from most sites. In contrast, the highest content was found in the samples from Cavallino-Treporti. - The Hg concentrations between two nearby sampling sites, one in the open sea and the other located in a lagoon, differed differed considerably, being six times as high in Cavallino-Treporti than in Caorle, confirming significant differences between lagoons and other sites	- The 72 pooled samples from sites along the Italian coasts were below the current maximum limits set by the European Commission.<br>- In the samples, the Hg levels were low, also in the specimens from polluted areas, probably due to their low-trophic level.<br>- A diet rich in seafood offers a variety of nutritional benefits, but it can also be a source of pernicious contaminants, such as metals, that pose a potential risk for human health.
		30	Monfalcone gulf		<0.01	0.31	0.12		
		30	Marano lagoon		0.2	0.26	0.24		
		44	Caorle open sea		<0.01	0.054	0.029		
		60	Cavallino treporti lagoon		0.014	0.34	0.20		
		30	Chioggia Lagoon		0.072	0.08	0.078		
		60	Caleri lagoon		<0.01	<0.01	<0.01		
		30	P. Garibaldi open sea		<0.01	<0.01	<0.01		
		30	Cervia open sea		<0.01	<0.01	<0.01		
		30	Giulianova Harbour		<0.01	<0.01	<0.01		
		60	Capoaiale-Varano lagoon		<0.01	<0.01	<0.01		
		30	Orbetello lagoon		0.22	0.29	0.25		
**Cabañero Al. 2005 (67)**	*Trachurus trachurus*		Sesimbra, Portugal)				0.033	- Mercury concentrations were highest in swordfish and tuna, and significantly lower in sardine, mackerel shad, and octopus.<br>- Speciation revealed that 93–98% of total mercury in fish samples was in the organic form of methylmercury (MeHg).	- All mercury concentrations were within the maximum limits set by European legislation.<br>- More than 93% of the total Hg occurring in fish samples was methylmercury.
**Cirillo T. 2010 (32)**	10 different species		Markets and stores from the main cities of Campania (Italy),					- All of the fresh catch and frozen fish species and 99% of the aquaculture fish in the study showed detectable amounts of Hg	- Mean methylmercury estimated intake does not exceed the safety limit recommended for this organic form, 1.6 mg/kg bw/week.
**Cossa D. 2012 (30)**	*Merluccius merluccius*	71	Bay of Biscay		0.04	0.66	0.30	The shelf edge and deep sediments may be a more significant source of MeHg than continental shelf sediment	- Within the Mediterranean hake population, a substantial increase in biomagnification power was observed as individuals reached adulthood, resulting from the combined effects of a reduced growth rate and changes in feeding habits during their life cycle.<br>- Hg concentrations, were highest in hake from the edge of the shelf and lowest in hake from the Rhone pro-delta area, suggesting lower bioavailability of MeHg in coastal environments.
		442	Gulf of lyon		0.11	14.31	1.67		
**Costa F. 2021 (78)**	*Trachurus trachurus*	69	Portuguese coast	muscl e	0.018	0.34	0.11	- Total mercury accumulation in tissues followed the order: muscle > liver > heart > gills > brain. All T-Hg concentrations were below the European regulatory limit.<br>- A strong, positive correlation was observed between fish age and T-Hg concentrations across all tissues, indicating a continuous bioaccumulation process and exponential growth over the lifespan.<br>- Methylmercury constituted, on average, 80% of total *mercury in muscle tissue.*	- Noteworthy importance of consumer options, such as the choice of smaller sized individuals, to minimize the risks associated with fish consumption.<br>- Risk assessment, based on (PTWI), indicated approximately 30% of the samples, specifically older and larger fish, exceeded the limit for MeHg, assuming average Portuguese fish consumption.
**Damiano S. 2011 (25)**	*Xiphias gladius.*	12	Ionian sea	meat			1.58	- Fish consumption is the primary source of heavy metals for man.<br>- There has not been any evidence of poisoning effects due to the intake of metals from the consumption of fish.	- Therefore, while concentrations of heavy metals such as Cd, Pb, and Hg do not represent a serious problem for human consumption, fish resources are still definitely at risk.
		10	Southern Tyrrhenian sea	meat			2.41		
		12	Central Tyrrhenian sea	meat			1.04		

		11	North-western Atlantic	meat			0.88		
		11	North-central Atlantic	meat			0.66		
**Di Bella C. 2020 (28)**	*Pagellus erythrinus*	28	Sicily market or caught	meat	0.94	1.45	1.13	- The higher Hg concentrations measured in seafood samples is probably related to ecological habits of species.	- The evaluation of human health risk related to seafood products consumption evidenced the overcoming of (PTWI) for Hg recommended by the European Food Safety Authority and WHO, and a non-carcinogenic risk (THQ) for Hg intakes occurs in baby, children and teenagers
	Pagellus acarne	8		meat	0.52	0.67	0.59		
	Pagellus bogaraveo	1		meat	0.06	0.06	0.006		
	Mullus barbatus	6		meat	1.71	2.11	1.91		
	Diplodus annularis	2		meat	3.51	/	/		
	Diplodus sargus	2		meat	0.48	/	/		
	Trigla lucerna	2		meat	0.66	/	/		
	Sphyraena sphyraena	1		meat	0.78	/	/		
	Sepia officinalis	7		meat	0.07	0.78	0.21		
	Penaeus kerathurus	39		meat	0.56	0.62	0.21		
	Pagellus acarne	8		meat	0.52	0.67	0.59		
	Pagellus bogaraveo	1		meat	0.06	0.06	0.006		
	Mullus barbatus	6		meat	1.71	2.11	1.91		
	Diplodus annularis	2		meat	3.51	/	/		
	Diplodus sargus	2		meat	0.48	/	/		
	Trigla lucerna	2		meat	0.66	/	/		
	Sphyraena sphyraena	1		meat	0.78	/	/		
	Sepia officinalis	7		meat	0.07	0.78	0.21		
	Penaeus kerathurus	39		meat	0.56	0.62	0.21		
**Di Bella G. 2015 (41)**	*Thunnus thynnus M*	13	Mediterranean Sea	muscle	0.25	0.714	0.40	- Determined trace element levels in the muscle, eggs, and sperm of 23 Thunnus thynnus specimens collected from the Mediterranean Sea.<br>- No tested tuna muscle sample exceeded the European maximum limits for Hg.	- Ingestion of toxic elements studied from tuna fish samples did not present any health risk for the average consumer.<br>- The regular or excessive consumption of tuna fish species might exceed the recommended (PTWI).
	Thunnus thynnus F	10	Mediterranean Sea	muscl e	0.278	0.647	0.45		
**Di Lena G. 2017 (21)**	*Chelidonichthys lucerna*	28	Central Adriatic Sea (CA)	Muscle	N.R	N.R	0.24	The highest mean total mercury level found in this study g well above the maximum level of 1.0 mg k as set by EU legislation, was found in large-size specimen (0.9-3.1 kg body weight) of angler (L. piscatorius) from CT, a carnivorous, long-living, high-trophic level bathy demersal species, inhabiting sandy and muddy bottoms.<br>- The mean total mercury levels obtained in this study ranged from 0.025 mg kg-1 to as high as 2.20 mg kg lowest levels in M. cephalus and L. ramada 0.038-0.081 mg/kg. Comparison between ca and ct: For steenbras, sardine, angler and fish drum, mercury levels were significantly higher in fish from CT than CA. In all other cases specimen recruited from CA belonged to a size-class significantly lower compared to specimen from<br>- The lowest levels were detected in tilapia (Oreochromis niloticus), a freshwater herbivorous species, while the highest were found in meagre (Argyrosomus regius), a marine species emerging in Mediterranean aquaculture, characterized by carnivorous dietary habit and high growth rate.<br>- The low extent of mercury accumulation in fish from intensive aquaculture is in accordance with the high growth rate of fish,	The majority of species sampled, demersal and pelagic-neritic, showed low mercury levels, regardless of their geographical origin. These species deserve constant monitoring studies and deep investigations on mercury accumulation, especially at the large size-classes. (Group4-5-6).<br>-On intensively farmed fish evidenced their low mercury contents, while levels of concern were found in seabass and seabream from the Lagoon of Orbetello.<br>- Analytical data showed mercury contamination levels of fish from the areas under study comparable to those reported for other Mediterranean areas, as emerge from the comparison with other studies
	Chelidonichthys lucerna	4	CentralTyrrhenia n Sea (CT)	Muscle	N.R	N.R	0.78		
	Dicentrarchus labrax	10	CentralTyrrhenia n Sea (CT)	Muscle	N.R	N.R	0.141		
	Dicentrarchus labrax	2	Central Adriatic Sea (CA)	Muscle	N.R	N.R	0.10		
	Diplodus puntazzo	6	CentralTyrrhenia n Sea (CT)	Muscle	N.R	N.R	0.09		
	Diplodus sargus	8	CentralTyrrhenia n Sea (CT)	Muscle	N.R	N.R	0.22		
	Diplodus vulgaris	6	CentralTyrrhenia n Sea (CT)	Muscle	N.R	N.R	0.23		
	Gobius paganellus	20	Central Adriatic Sea (CA)	Muscle	N.R	N.R	0.05		
	Litognatus mormyrus	17	CentralTyrrhenia n Sea (CT)	Muscle	N.R	N.R	0.13		
	Litognatus mormyrus	10	Central Adriatic Sea (CA)	Muscle	N.R	N.R	0.07		

Liza ramada	3	Central Adriatic Sea (CA)	Muscle	N.R	N.R	0.04
Merluccius merluccius	8	CentralTyrrhenian Sea (CT)	Muscle	N.R	N.R	0.08
Merluccius merluccius	16	Central Adriatic Sea (CA)	Muscle	N.R	N.R	0.04
Microchirus ocellatus	9	Central Adriatic Sea (CA)	Muscle	N.R	N.R	0.13
Microchirus variegatus	20	Central Adriatic Sea (CA)	Muscle	N.R	N.R	0.66
Mugil cephalus	3	Central Adriatic Sea (CA)	Muscle	N.R	N.R	0.04
Mugil cephalus	2	CentralTyrrhenian Sea (CT)	Muscle	N.R	N.R	0.02
Mullus barbatus	58	CentralTyrrhenian Sea (CT)	Muscle	N.R	N.R	0.70
Mullus barbatus	25	Central Adriatic Sea (CA)	Muscle	N.R	N.R	0.24
Mullus surmuletus	45	CentralTyrrhenian Sea (CT)	Muscle	N.R	N.R	0.13
Mustelus mustelus	2	Central Adriatic Sea (CA)	Muscle	N.R	N.R	0.93
Pagellus bogaraveo	5	CentralTyrrhenian Sea (CT)	Muscle	N.R	N.R	0.20
Platichthys flesus	10	Central Adriatic Sea (CA)	Muscle	N.R	N.R	0.04
Psetta maxima	4	Central Adriatic Sea (CA)	Muscle	N.R	N.R	0.08
Raja asterias	4	Central Adriatic Sea (CA)	Muscle	N.R	N.R	0.11
Sciaena umbra	2	Central Adriatic Sea (CA)	Muscle	N.R	N.R	0.12
Scorpaena scrofa	3	Central Adriatic Sea (CA)	Muscle	N.R	N.R	0.34
Scorpaena scrofa	8	CentralTyrrhenian Sea (CT)	Muscle	N.R	N.R	0.37
Scyliorhinus canicula	18	Central Adriatic Sea (CA)	Muscle	N.R	N.R	0.82
Solea vulgaris	19	Central Adriatic Sea (CA)	Muscle	N.R	N.R	0.025
Solea vulgaris	11	CentralTyrrhenian Sea (CT)	Muscle	N.R	N.R	0.081
Sparus aurata	4	Central Adriatic Sea (CA)	Muscle	N.R	N.R	0.24
Sparus aurata	6	CentralTyrrhenian Sea (CT)	Muscle	N.R	N.R	0.17
Trachinus draco	26	Central Adriatic Sea (CA)	Muscle	N.R	N.R	0.52
Umbrina cirrosa	3	Central Adriatic Sea (CA)	Muscle	N.R	N.R	0.035
Umbrina cirrosa	6	CentralTyrrhenian Sea (CT)	Muscle	N.R	N.R	0.083
Auxis rochei	2	Central Adriatic Sea (CA)	Muscle	N.R	N.R	0.28
Engraulis encrasicolus	120	Central Adriatic Sea (CA)	Muscle	N.R	N.R	0.041

reducing the exposition to contaminants and to the controlled feeding conditions

- A very low risk for human health is estimated for the consumption of fish that showed very low total mercury levels: Eating fish belonging to this group, within nutrients and energy needs, poses no risk to human health as regards mercury content (Group 1). Farmed tilapia, trout, seabass and gilthead seabream, tench reared in ponds and gilthead seabream from the lagoon of Venice.
- Level corresponds to a safe consumption of 3-4 portions per week range 0.13-0.20 mg kg-1 (Group 2) were demersal or pelagic-neritic species at a higher trophic level when compared to Group 1. Fish attaining mercury levels within the range 0.22-0.30 mg kg-1 (Group 3) provides 33-45 µg total mercury per 150 g-edible portion.
- From CA: Fish from group 4, contains 0.31-0.60 mg kg-1 of mercury, which corresponds to 46-91 µg per 150 g-edible portion, may be safely consumed once a week as the consumption of a portion provides a 70 kg-person 56-100% of the TWI of mercury.

-Group 5 within the range 0.62-1.21 mg kg-1 provides twice as much, corresponding to 101-198% TWI, resulting in safe consumption of only 2 portions per month. This group is represented by demersal and benthopelagic predatory species, most of them living in close contact with the sea bottom from

- Very high levels of mercury found in large-size specimens of L. piscatorius sampled in CT suggest a prudent consumption of no more than one single portion per month. In fact, the amount of mercury provided by one portion of angler, at the size sampled in this study, largely exceeds the TWI for a person weighing 70 kg (364% TWI).

Engraulis encrasicolus	81	CentralTyrrhenian Sea (CT)	Muscle	N.R	N.R	0.081
Sarda sarda	2	CentralTyrrhenian Sea (CT)	Muscle	N.R	N.R	0.802
Sardina pilchardus	45	Central Adriatic Sea (CA)	Muscle	N.R	N.R	0.038
Sardina pilchardus	90	CentralTyrrhenian Sea (CT)	Muscle	N.R	N.R	0.067
Scomber japonicus	23	CentralTyrrhenian Sea (CT)	Muscle	N.R	N.R	0.17
Scomber scombrus	28	Central Adriatic Sea (CA)	Muscle	N.R	N.R	0.046
Scomber scombrus	8	CentralTyrrhenian Sea (CT)	Muscle	N.R	N.R	0.52
Seriola dumerili	6	CentralTyrrhenian Sea (CT)	Muscle	N.R	N.R	0.049
Sprattus sprattus	120	Central Adriatic Sea (CA)	Muscle	N.R	N.R	0.059
Thunnus thynnus	7	South Tyrrehenian Sea (ST)	Muscle	N.R	N.R	0.81
Lepidopus caudatus	3	CentralTyrrhenian Sea (CT)	Muscle	N.R	N.R	1.17
Merlangius merlangus	27	Central Adriatic Sea (CA)	Muscle	N.R	N.R	0.12
Pagellus erythrinus	5	CentralTyrrhenian Sea (CT)	Muscle	N.R	N.R	0.44
Pagellus erythrinus	41	Central Adriatic Sea (CA)	Muscle	N.R	N.R	0.89
Phycis blennoides	9	CentralTyrrhenian Sea (CT)	Muscle	N.R	N.R	0.72
Trisopterus minutus	12	Central Adriatic Sea (CA)	Muscle	N.R	N.R	0.10
Zeus faber	5	CentralTyrrhenian Sea (CT)	Muscle	N.R	N.R	0.75
Zeus faber	2	Central Adriatic Sea (CA)	Muscle	N.R	N.R	0.26
Lophius piscatorius	3	CentralTyrrhenian Sea (CT)	Muscle	N.R	N.D.	2.20
Lophius piscatorius	3	Central Adriatic Sea (CA)	Muscle	N.R	N.R	0.37
Oreochromis niloticus	2	Intensive	Muscle	N.R	N.R	0.007
Onchorhynchus mykiss	5	Intensive	Muscle	N.R	N.R	0.014
Dicentrarchus labrax	3	Exstensive lagoon Venice	Muscle	N.R	N.R	0.23
Dicentrarchus labrax	5	Exstensive lagoon Orbetello	Muscle	N.R	N.R	0.89
Dicentrarchus labrax	4	Intensive	Muscle	N.R	N.R	0.043
Sparus aurata	6	Intensive	Muscle	N.R	N.R	0.044
Sparus aurata	3	Exstensive lagoon Venice	Muscle	N.R	N.R	0.07
Sparus aurata	4	Exstensive lagoon Orbetello	Muscle	N.R	N.R	0.67

	Tinca tinca	5	Semi intensive	Muscle	N.R	N.R	0.047		
	Argyrosomus	4		Muscle	N.R	N.R	0.251		
**Di Lena G. 2018 (54)**	*Aristeus antennatus*	28 (CT)	Central Tyrrhenian Sea (CT)	Abdomen	0.18	1.054	0.54	- A a higher contamination of the Tyrrhenian site, a fact we also observed on fish species (Squilla mantis).<br>- No risk is envisaged at consumption of caramote prawn (Penaeus kerathurus), warty crab (Eriphia verrucosa) and European spider crab (Maja squinado) attaining mercury levels <0.25 mg/k. Norway lobster (N. norvegicus) and mantis shrimp from the CT site may be safely consumed in the amount of 2–3 portions per week by a 70-kg person. Deep-water rose shrimp (P. longirostris) and blue and red shrimp (A. antennatus), due to their higher mercury levels, may be safely consumed once a week, since a 100 g-portion of these species provides a 70-kg person 50–80% the TWI. Norway lobster (N. norvegicus) sampled in the CA site attained mercury levels not safe for consumption	- Crustacean species living in coastal areas in sandy or rocky (P. kerathurus, E. verrucosa, M. squinado) bottoms showed a low mercury accumulation compared to species living at a high depth, in close contact with muddy bottoms. For shrimps like P. longirostris, A. antennatus and P. kerathurus, the inter-individual variability observed was mostly related to size, indicating an accumulation of mercury with age. For crabs and lobsters, no direct relation between body size and mercury levels was recorded.
	Parapenaeus longirostris	72	Central Tyrrhenian Sea (CT)	Abdomen	0.24	0.702	0.46		
	Penaeus kerathurus	107	Central Tyrrhenian Sea (CT) n. 43 (20F-23M)	Abdomen	0.054	0.27	0.76		
	Penaeus kerathurus		Central Adriatic Sea; (CA) n. 64 (20F-44M)	Abdomen	0.024	0.13	0.072		
	Nephrops norvegicus	85	CentralTyrrhenian Sea (CT) n. 35	Abdomen	0.13	0.611	0.29		
	Nephrops norvegicus		Central Adriatic Sea; (CA) n. 50	Abdomen	0.54	2.34	0.68		
	Palinurus elephas	4	CentralTyrrhenian Sea (CT)	Abdomen	0.11	1.07	0.36		
	Squilla mantis	45	CentralTyrrhenian Sea (CT) N.21	Abdomen	N.R	N.R	0.41		
	Squilla mantis		Central Adriatic Sea; (CA) n. 24	Abdomen	0.03	0.20	0.08		
	Eriphia verrucosa	17	Central Adriatic Sea (CA)	Cephalotorax white meat	0.058	0.25	0.028		
	Maja squinado	11	Central Adriatic Sea (CA)	Cephalotorax white meat	0.076	0.51	0.16		
**Di Leo A. 2010 (57)**	*Mytilus galloprovincialis Lam.)*	7 stations	Mar Piccolo of Taranto	mussels tissue			0.39	- THg and MeHg levels in muscles analyzed from the Mar Piccolo of Taranto, finding concentrations ranging from 0.236-0.559 µg/g d.w.<br>- Values were generally below EU limits for mussels and the PTWI for adults, children in Taranto were near the THg intake limit, especially when consuming mussels from more contaminated areas.<br>- The Mar Piccolo was found to be a mercury-polluted environment, with health risks not being excluded for high-level consumers or vulnerable populations like children and pregnant/lactating women.	- The EWI of THg and Me-Hg were always below the established PTWI for all sampled mussels, although, in relation to the average body weight, intake by children of THg was near the PTWI especially using the concentrations registered in mussels collected in stations 1 and 7.<br>- Health risks due to the dietary THg intake for children, especially for fisherman's son of Taranto population, cannot be excluded.<br>- Pregnant and lactating women should pay particular attention to the amount of seafood introduced in their diets.
	Mytilus galloprovincialis Lam.)	3 stations		mussels tissue			0.30		
**Djermanovic M. 2020 (68)**	*Salmo salar* Frozen	8	market of the Republic of Srpska Bosnia and Herzegovina	muscle	0.012	0.026	0.017	- In summary, mercury levels in fish available in the market of the Republic of Srpska, Bosnia and Herzegovina were lower in comparison to those found in similar national and international studies	- The average concentrations of mercury found in all species analyzed were below the limits set by the European and Bosnian and Herzegovinian legislation. - A surveillance system of mercury content in fishery products, especially in certain species that concentrate mercury, is crucial for public health protection.
	Macruronus magellanicus Frozen	26		muscle	0.018	0.048	0.033		
	Scomber scombrus Frozen	4		muscle	0.008	0.018	0.012		
	Sprattus sprattus Frozen	9		muscle	0.012	0.005	0.007		

	Pangasius hipophthalmus Frozen	11		muscle	0.005	0.154	0.59		
	Thunnus albacores canned	28		muscle	0.005	0.094	0,054		
	Sprattus sprattus Canned	11		muscle	0.01	0.084	0,031		
	Scomber scombrus canned	8		muscle	0.014	0.094	0,025		
**Ervik H. 2018 (80)**	*Cancer pagurus*	66	Norway	Brown meat	0.02	0.76	0.12	- In the region Mausund, there were between year (2012–2015) variations in some concentrations of toxic or essential elements in edible crab and cod, but no variation in halibut. In brown meat in crab and for the elements B, Sn, Cs, Hg, Cr, and As<br>- In cod, there were significant differences between 2012 and 2015 for the elements As, B, Cr, Cs, Hg, and Sn and there were significant differences between locations for the elements B and Pb	- Comparing the concentrations of toxic or essential elements in edible crab and halibut, caught at two different locations within the region, cod caught at five locations within the region and sediment sampled at three locations within the region, showed that except for the concentrations of Sn in brown meat in crab and B and Pb in cod, there appeared to be no difference between the location close to a fish farm and the other locations.
	Gadus morhua	50		muscle	0.034	3.15	0.56		
	Hippoglossus hippoglossus	9		muscle	0.038	0.60	0.27		
**Esposito M. 2018 (42)**	*Xiphias gladius*	20	Mediterranean Sea	muscle	0.12	1.66	0.61	- About 50% of the total alerts for mercury are all attributed to the swordfish, giving therefore strong relevance to the relationship Hg intake-swordfish.<br>- Muscle is considered the main reservoir for the accumulation of contaminants.	-The swordfish is a good "reservoir" of mercury and suggests that the possible exposure risk due to Hg in swordfish should be considered for some groups of consumers like children.
	Xiphias gladius	27	Nord East Atlantic Ocean	muscle	0.033	2.14	0.47		
**Faganeli J. 2018 (90)**			Gulf of Trieste (Northern Adriatic Sea)					- The molar Hg/Se ratios in seawater plankton, and sediment were <0.5, while those in particulate matter were <1.3. In benthic ray species, a parallel increase in Se and Hg in muscle was observed, indicating that increased Hg (MeHg) bioaccumulation results in Se coaccumulation	- It appears that Se in apex predators in the Gulf of Trieste, characterized by rather high seawater Se levels, moderates the bioaccumulation and environmental toxicity of Hg at levels higher than those set by EU food safety legislation.
**García MÁ. 2016 (43)**	*Thunnus albacores* FRESH	10	Central Market of Lugo (Galicia, NW Spain	muscle	0.557	1.07	0.76	- 110 samples analyzed of fresh and processed tuna marketed in Galicia, finding mercury (Hg) present in all samples. Only one fresh tuna sample exceeded the EU maximum limit for predatory fish.<br>- Average mercury concentrations in processed tuna are significantly lower than in fresh tuna. For canned tuna, the Hg content followed the order: olive oil > natural > pickled sauce, with statistically significant differences for pickled sauce.<br>- No influence of packaging type (glass or can) on tuna mercury content was observed	I confirmded the widespread presence of mercury in tuna, with only a single fresh tuna sample marginally exceeding EU legal limits.<br>It was clearly demonstrated that mercury concentrations are higher in fresh tuna compared to processed tuna and that albacore. Although current levels are considered safe for average consumer health according to existing regulations.
	Thunnus albacores CANNED	90	major supermarkets of the city of Lugo (Galicia, NW Spain)	muscle	0.08	0.715	0.30		
	Thunnus albacores PRESERVED IN GLASS	10	major supermarkets of the city of Lugo	muscle	0.095	0.558	0.31		
**Gutiérrez AJ. 2005 (59)**	*Mollusca, Bivalvia Tinned*	120	Galician conserve industries (northwestern Spain) or Chile.		0.011	0.063	0.027	- All mercury concentrations were found to be below the maximum permitted limit for human consumption, as defined by EU.<br>- Mean mercury concentrations were: cockles 66.59 ± 23.53 µg/kg, variegated scallops 33.68 ± 15.76 µg/kg, mussels 27.83 ± 12.43 µg/kg, and razor shells 21.26 ± 12.24 µg/kg.<br>- Cockles exhibited the highest concentration, which was unexpected considering their small size.<br>- These levels were comparable to or lower than those reported in other European countries	- A person would have to ingest 17,966.22 g of mussels, 7,508.63 g of cockles, 14,846.61 g of variegated scallops, and 23,518.30 g of razor shells to reach the ADI.
	Cerastoderma edule	40			0.025	0.10	0.066		
	Chlamys varia	24			0.008	0.044	0.033		
	Ensis spp	36			0.001	0.057	0.021		
		16					0.23		
		16					0.36		
		16					0.086		
		16					NONE		
		16					0.445		
		16					0.483		
**Harmelin-Vivien M. 2009 (27)**	*mullus barbatus*	132	Gulf of lyon		0.02	8.92	1.11	- No significant relationship between Hg concentration and size and age was observed in M. surmuletus and M. b. ponticus	- Mercury concentration in fish muscle was six times higher in the two Mediterranean species than in the Black Sea one for similar sized animals.
	mullus surmuletus	85			0.001	6.06	0.92		

									- The concentration of bioavailable Hg in seawater is incapable to satisfactorily explaining Hg variations in marine biota.
**Harmelin-Vivien M. 2012 (53)**	*Merluccius merluccius*		gulf of lyon					- Organic contaminants exhibited also a trend to increased concentrations with fish size	- The study concluded that European hake in the Northwestern Mediterranean (Gulf of Lions) exhibits higher mercury concentrations compared to Atlantic populations.<br>- Lower mercury bioavailability in coastal environments compared to offshore areas.
**Horvat M. 2014 (39)**	*Myliobatis aquila*	5		muscle	0.051	0.18	0.086	- Hg and MeHg contents were positively correlated with the size/age of bull ray tissues.<br>- The highest percentage of mercury as MeHg in all ray species was found in muscle tissue	- MeHg bioaccumulation between sediment and benthic invertebrates and the muscle of small and of larger benthic fish ranged between 102 and 103, suggesting greater accumulation by pelagic feeding species.<br>- MeHg, originating mostly from sedimentary production and encountered in higher trophic levels in this area including fish, demonstrates dietary bioaccumulation.
	Pteromylaeus bovinus	17		muscle	0.08	1.79	1.028		
	Dasyatis violacea	8		muscle	0.49	1.344	0.871		
	Dasyatis pastinaca	1		muscle	0.4	/	/		
**Junqué E. 2018 (56)**	22 Different species	Western Mediterranean Sea - nearby balearic islands						- 39% of the specimens from the Mediterranean, excluding angler, have Hg concentrations above the EU threshold of 0.5 μg/g ww	- The Hg concentrations were significantly higher in the dusky grouper specimens from the Mediterranean Sea than from the Atlantic Ocean.<br>– 41% of the specimens from the Mediterranean and 25% of the species from the Atlantic Ocean analysed in the present study showed Hg concentrations above the EU recommended limit for human consumption.
**Jędruch A. 2019 (63)**	*Mytilus trossulus*	99	Baltc Seas				0.029	-The obtained results showed that the HgTOT concentration was primarily shaped by the individual features of specimens, related to the growth and reproductive cycle of mussels, as well as the quality of food consumed by them.<br>-A positive relationship was noted between the HgTOT concentration in a mussel's tissues and the length of its shell. The total share of labile Hg forms in *M. trossulus* was high, exceeding 90% of HgTOT.	- Levels of HgTOT and organic Hg deemed to be safe for humans.<br>HgTOT concentration accounted for about 6% of the maximum permissible level of Hg in seafood.<br>- The biomagnification factor (BMF), which accounted for the trophic position of *M. trossulus*, indicated a 3-fold increase in HgTOT concentration on each subsequent food level, suggesting an important role played by this species in the transfer of Hg along the marine food chain.
**Juresa D. 2003 (47)**	*Pitaria chione*	3	Croatian coast at seven locations of the Adriatic Sea were purchased at Zagreb fish market	Muscle			0.13	- The EWI of total mercury from fish and shellfish was in the range 0.02–0.07 mg, representing 6–20% of the PTWI.	- The concentrations of toxic trace elements of mercury, […] in seafood are similar in the whole area of the Adriatic Sea.<br>- There are strong arguments for probabilistic assessments to be undertaken using the available data. Because of the high variability in trace elements' content in seafood Total mercury concentration in hake was directly correlated with the length of specimen
	Mytilus galloprovincialis	10					0.18		
	Scomber scombrus	3					0.15		
	Scomber scombrus	3					0.21		
	Trachurus trachurus	3					0.21		
	Boops boops	3					0.19		
	Sardina pilchardus	10					0.14		
	Sardina pilchardus	10					0.20		
	Engraulis encrasicholus	10					0.15		
	Merluccius merluccius	3					0.27		
	Merluccius merluccius	3					0.37		
**Kammann U. 2022 (81)**	*limanda limanda*							- THg concentrations in dab muscle significantly increased by 1.4% annually, leading to a 41% rise in contamination levels over a 25-year period, a trend found to be independent of fish age.<br>-THg concentrations in nearby North Sea sediments significantly decreased during the same period, indicating that sediment trends cannot explain the increase observed in fish.	- There is a contradiction between contamination trends in biota and sediment, emphasizing the importance of simultaneously investigating both environmental compartments for accurate monitoring and risk assessment
	Thunnus thynnus	18		gills	0.32	0.80	0.51		

**Kljaković-Gašpić Z. 2021 (23)**			open waters of the central Adriatic Sea	white muscles behind the head	0.92	2.37	1.4	- THg concentrations in all tissues and Se in liver and caudal muscle were positively correlated with tuna age and size.<br>- Founded significant differences in total mercury (THg) and selenium (Se) levels, and their molar ratios (Se:THg) among different tissues (white and red muscles, liver, gills) of wild Atlantic bluefin tuna (ABFT) from the central Adriatic Sea. THg and Se levels were highest in the liver, with THg lowest in gills and Se lowest in white muscle.	- Concentrations of THg and Se significantly differed between the analyzed tissues.<br>- THg levels were highest in liver, intermediate in muscles and lowest in gills. THg concentrations in red muscle were significantly higher in comparison to different white muscle cuts (liver > red muscle > all white muscle cuts > gills).
				white muscle's middle dorsal part	0.76	2.48	1.32		
				white muscles Tail	0.87	2.72	1.39		
				red muscles from the middle part	1.13	3.89	1.89		
**Knowles T.G. 2003 (82)**	*Hippoglossus hippoglossus*	8			0.038	0.617	0.29	- The highest mercury levels were found in billfish and shark. Specifically, all 5 shark samples and 13 out of 20 billfish samples exceeded their respective European Commission limit.<br>- One fresh tuna sample (out of 20) also exceeded its limit, but canned tuna concentrations were, on average, half that of fresh/frozen tuna.<br>- In contrast, mercury concentrations in UK-farmed salmon and trout were relatively low. Overall, 20 out of the 336 samples examined exceeded the maximum permitted levels, primarily among imported predatory species	- Levels of mercury in the vast majority of samples and in all the more commonly consumed products were within EC limits.<br>- Most results found to be above the relevant limits were in samples of imported fresh fish (predatory fish) In particular, mercury levels in billfish (swordfish and marlin) and shark (species unknown) were frequently above the maximum permitted levels.
	Macruronus magellanicus	2			0.065	0.307	0.18		
	Lophius piscatorius	2			0.096	0.3	0.19		
	Hoplostethus atlanticus	6			0.52	0.647	9.55		
	OTHER	12			0.006	0.66	0.10		
	Pollachius pollachius	4			0.007	0.02	0.012		
	Salmo salar	14			0.029	0.079	0.05		
	Dicentrarchus labrax	4			0.03	0.094	0.065		
	Sparus aurata	4			0.051	0.056	0.053		
	Selachimorpha	5			1.006	2.2	1.52		
	Istiompax indica	20			0.153	2.70	1.34		
	Salmo trutta	14			0.014	0.10	0.06		
	Thunnus thynnus	20			0.141	1.5	0.401		
	Exotic prawns	14			0.006	0.047	0.025		
	Nephropidae Dana	4			0.009	0.231	0.075		
	mussels	4			0.017	0.041	0.03		
	OTHER	9			0.003	0.186	0.038		
	prawns	14			0.013	0.249	0.048		
	squid	9			0.003	0.036	0.011		
	OTHER	9			0.009	0.06	0.032		
	paste and pate´s	9			0.019	0.166	0.068		
	other smoked	9			0.010	0.932	0.307		
	Salmo salar smoked	9			0.037	0.081	0.055		
	trout smoked	9			0.052	0.09	0.071		
	anchovy canned	9			0.028	0.064	0.049		
	OTHER canned	9			0.003	0.078	0.024		

	Sardina pilchardus canned	9			0.005	0.047	0.019		
	salmon canned pink	19			0.008	0.042	0.028		
	salmon canned red	13			0.012	0.066	0.036		
	sardine	9			0.012	0.104	0.041		
	Thunnus thynnus canned	54			0.031	0.71	0.19		
**Licata P. 2004 (20)**	*Thunnus thynnus*	5	Straits of Messina				3.37	- Determined heavy metal levels (Cd, Cu, Hg, Mn, Pb, and Zn) in liver and muscle tissues of *Thunnus thynnus* collected from the Straits of Messina.<br>- Significant variations () for Hg concentrations in muscle than in liver samples. The highest concentrations of Hg ()were found in muscle,<br>- Negative correlation between mercury level and weight and length in samples from both female and male tuna.	-Levels of Hg are above the MRLs (1 \mu g/g) in muscle, thereby establishing toxicological risks for the consumer. -- *Thunnus thynnus* tends to have higher mercury concentrations due to its location at higher trophic levels and its high capability of bioaccumulation and biomagnification.<br>-The mercury levels found in these Mediterranean tuna samples are significantly higher than those reported in Atlantic tuna, possibly due to pollution and under-water volcanism.
**Llull RM. 2017 (19)**	*Engraulis encrasicolus*	7	Balearic Islands and Tunisia Egypt	Meat	0.05	0.1	0.057	- Were determined the concentrations of THg and MeHg in 32 different lean fish species from the Western Mediterranean Sea. with a special focus on the Balearic Islands.<br>- Two studies performed in Italy on specific fish species found higher EWIs than in the present study. However. these Italian studies used mean THg values whereas median values were used in the present study. which tend to generate lower estimations as fish mercury distributions tail towards high values-	- The estimated intake of total mercury from Western Mediterranean fish is generally below recommended limits for most of the population, the intake of MeHg, significantly exceeds the PTWIs for both children and adults (190% and 150% respectively), even as maximum potential estimates. This highlights a potential health risk, especially for populations with high local fish consumption.
	Sardinella aurita	1			0.11	0.11	0.11		
	Spicara smaris	7			0.05	0.15	0.064		
	Aphia minuta	4			0.05	0.05	0.05		
	Sardina pilchardus	8			0.05	0.05	0.05		
	Spandyliosoma cantharus	14			0.05	0.79	0.19		
	Sciaena umbra	8			0.05	0.23	0.094		
	Scyliorhinus canicula	8			0.39	1.50	0.78		
	Pagellus bogavareo	2			0.11	0.3	0.21		
	Mullus surmuletus	9			0.05	0.49	0.18		
	Pagrus pagrus	12			0.05	1	0.31		
	Pagellus erythrinus	10			0.22	0.86	0.35		
	Solea solea	8			0.05	1.2	0.45		
	Xyrichtys novacula	7			0.05	0.13	0.061		
	Diplodus sargus	9			0.16	0.63	0.34		
	Serranus cabrilla	11			0.05	0.53	0.3		
	Trachurus trachurus	7			0.05	0.4	0.18		
	Serranus scriba	7			0.2	0.46	0.28		
	Epinephelus marginatus	10			0.57	3	1.60		
	Scorpaena scrofa	35			0.05	0.58	0.22		
	Conger conger	31			0.17	1.8	0.56		
	Dentex dentex	17			0.15	1.5	0.85		
	Scorpaena porcus	2			0.15	0.16	0.16		
	Sphyraena sphyraena	1			1	1	1		
	Zeus faber	16			0.05	1.3	0.33		
	Copyphaena hippurus	3			0.05	0.05	0.05		

	Merluccius merluccius	31			0.05	0.9	0.3		
	Lamna nasus	1			3	3	3		
	Muraena helena	38			0.24	0.68	0.42		
	Lophius piscatorius	34			0.12	3.1	0.74		
	Seriola dumerili	16			0.05	1.9	0.23		
**Lozano-Bilbao E. 2023 (50)**	*Scomber colias*	90	Isole Tenerife (Isole Canarie)					- Significant differences in mercury concentrations between the sampling years. with a clear decreasing trend over the decades.	- The decadal decreasing observed in the mercury content in S. colias here presented could be explained by the strict application of toxic metal control protocols in recent decades
**Lundebye AK. 2017 (73)**	*Salmo salar wild*	87	Norwegian coastal waters	Muscle	0.014	0.13	0.037	- Wild Atlantic salmon had significantly higher levels of contaminants including […] and mercury. compared to farmed salmon.<br>- Wild salmon also contained higher concentrations of essential elements like selenium. copper. zinc. and iron. and the marine omega-3 fatty acid DHA. than farmed salmon.	- The present study demonstrates that the concentrations of […] and mercury in wild Atlantic salmon were higher than in farmed Atlantic salmon.
	Salmo salar escaped farmed salmon	12					0.029		
	Farmed salmon	305			0.007	0.042	0.015		
**Magalhães. MC 2007 (69)**	*Pagellus acarne*				0.07	1.99	0.92	The MeHg was the major form of mercury accumulated in all species, comprising an average of 88.1% of total mercury.<br>- Mercury concentrations increased with fish age, length, and weight.<br>- A strong positive correlation was found between mercury levels in muscle and estimated mercury in the diet, with total Hg concentrations in muscle approximately nine times those estimated in food.<br>- Total mercury concentrations in muscle were also positively correlated with both trophic level and median depth	- Median total mercury concentrations obtained for all the analyzed species were below the established limit for safe human consumption.<br>- Necessity to include MeHg determinations in monitoring programs, particularly in the North Atlantic where, in recent years, the exploitation of deep-sea fish stocks has increased greatly.
	Phycis phycis		Azores		0.23	1.91	0.59		
	Trachurus picturatus				0.05	2.03	0.72		
	Lepidopus caudatus				0.23	3.2	1.44		
	Conger conger				0.24	5.24	1.86		
	Polyprion americanus				0.39	3.94	1.23		
	Phycis blennoides				0.38	1.07	0.69		
	Mora moro				0.51	11.74	3.7		
**Mancini L. 2022 (35)**	*Sediments*		Orbetello lagoon					- HgT concentrations in the sediments of the Orbetello lagoon exceeded the EQS of Italian legislation<br>- Hgtot concentrations in mussels. ranging from 0.050 to 0.324 mg/kg wet weight. did not exceed the European food legislation limits. However. all Hgtot values in mussels exceeded the EU environmental quality standard threshold for biota.	- This study revealed that the sediment contamination in the Orbetello lagoon can potentially affect the food chain in the area. with a possible risk to human health through the consumption of seafood.
	Mytilus Galloprovincialis	2kg			0.1	0.158	0.12		
		2kg			0.205	0.324	0.25		
		2kg			0.052	0.088	0.07		
		2kg			0.05	0.092	0.07		
**Mauffreta A. 2023 (79)**	Cod *(Gadus morhua),* Dogfish *(Scyliorhinus canicular),* Mackerel *(Scomber scombrus),* Plaice *(Pleuronectes platessa), Whiting (Merlangius merlangus),* Blue whiting *(Micromesistius poutassou),* Hake *(Merluccius merluccius),* Sardine *(Sardina pilchardus)*	8 species	Eastern English Channel, Bay of Biscay, Gulf of Lions (GoL) in Western Mediterranean Sea.	NA				- A limited influence of the fish habitat (pelagic, demersal, benthic), diet (zooplankton, piscivore) or trophic levels on NDL-PCB and TEQ concentrations (lp-basis). Further explorations of As speciation and bioavailability are needed to assess general tendencies regarding its behaviour in marine fish.	Contamination patterns in chondrichthyan clearly differed from that in teleost fish.<br>- Trophic levels were significantly correlated to contaminant concentrations only for several substances. Development of taxa-specific thresholds might be a practical way forward to refine environmental assessment.
**Merciai R. 2018 (51)**	*Diplodus sargus (L.)*	81	Gulf of lyon	muscle	0.044	2.27	0.64	- D. sargus, a demersal species feeding at direct contact with contaminated sediment, and ii) the relatively long lifespan of this species, comparable with the one of large predators such as tuna;	- The white seabream D. sargus may exhibit very high concentrations of Hg in its edible part, even in apparent absence of considerable contamination sources,

								the key to high levels of Hg bioaccumulation, therefore, would be age, i.e. the time of exposure to contaminants, rather than body size	representing a hazard for humans in spite of being poorly acknowledged as a species prone to accumulate this highly toxic heavy metal
**Bank MS. 2021 (83)**	Greenland halibut *(Reinhardtius hippoglossoides)*	625	Norwegian Sea, Norway.	NA				- The Hg concentration in molluscs was, thus, ranked as cephalopod [gastropod > bivalve. The Hg concentrations significantly differed among molluscs	- Elevated Hg concentrations were seen in cephalopods whereas the elevated activity concentrations of 210Po were noticed in bivalves, which was mainly elucidated with disparate feeding ecology. Total Hg in fillet tissue was predominantly in the MeHg form (>77 %).
**Miedico O. 2015 (38)**	16 Different species	305						- The higher mean contamination level related to mercury was detected in teleosts samples.<br>- There was no close correlation among lead, cadmium and mercury accumulation in mussels. Moreover, it is important to underline<br>that mercury accumulation relates to that of methylmercury, which is the most toxic form of mercury.	- High contamination by mercury related to swordfish was 36%. Another important result to underline is the not negligible contamination by mercury verified in blue-fish samples which are usually recommended in many diets
**Milatou N. 2020 (77)**	*Thunnus thynnus*	268	Greek tuna farm in the Ionian Sea		0.49	1.60	0.86	- Exposure of consumers can decrease their exposure to Hg preferring to eat bluefin tuna reared for a longer period. - - The rearing process decreases the mercury contents in fish. Thus, selecting fish species with low mercury concentrations to feed the reared bluefin tuna might decrease the mercury content of the reared specimens.	- Accumulation of Hg in the muscle tissue of reared Atlantic bluefin tuna is affected not only by the size and age of the fish, but it is also influenced to a remarkable degree by the rearing period.<br>- The rearing period in the sea cages, the lower the Hg concentrations in the muscle.
**Milatou N. 2023 (52)**	*Thunnus thynnus*	260	Greek tuna farm (at the Echinades Islands)				0.87	The few studies conducted till now on farmed fishes have mainly examined the effect of lipid content on mercury levels, as mercury is one of the most hazardous trace metals. noticed that Hg concentrations in muscle tissue of reared southern bluefin tuna, T. maccoyii, decreased with the increase of lipid content, regardless of the availability of protein binding sites in tissues. The results of the present study showed that 40 % of the muscle tissue samples contained Hg above the maximum level of 1 mg/kg w/w set by the European Commission Decision. Considering the Hg levels found, it could be deduced that people who consume this species regularly may expect serious health problems in the long term.	The results indicate that the accumulation of Hg and Zn in the muscle tissue of reared Atlantic bluefin tuna is influenced by the protein-lipid composition since statistically significant correlations (positive or negative) arose for the samples studied. Specifically, the higher lipid content is related to lower Hg levels, whereas the higher protein content is related to higher Hg levels
**Mille T. 2021 (5)**	*Merluccius merluccius*	32	Bay of Biscay	muscle	0.078	0.65	0.26	- All individuals. except four hakes. had THg concentrations below the food safety thresholds determined by the European commission regulation.<br>- MeHg concentrations were higher in the muscle than in the liver and the gonads for the three species.<br>- Hg concentrations in muscle were significantly lower in sole than in hake and red mullet	- The biomagnification of MeHg and thus the transfer of this Hg species in the trophic network, resulting in high MeHg concentration in a piscivorous fish species.<br>- The present study confirmed that MeHg represent the majority of Hg in fish, consistently with most literature records
	Mullus surmuletus	17			0.21	0.58	0.33		
	Solea solea	41			0.038	0.34	0.118		
**Millour S. 2011 (48)**	*FIsh*	46			0.05	0.70	0.065	- The levels observed in this study are generally similar to or lower than those observed in the data provided to EFSA by 12 EU Member States and Norway (36,651 results reported).	The levels are generally similar to or lower than those observed in the data provided to EFSA by 12 EU Member States and Norway Fish and fish products'' by , Hg, […]. Shellfish were generally more contaminated than fish by these contaminants, except in the case of Hg.
	Crustacean	37			0.005	0.04	0.019		
**Minganti V. 2010 (88)**	*Sparus aurata*	26	Farmed	meat	0.07	0.16	0.12	- Compared the concentrations of several trace elements in muscle tissue of farmed and wild gilthead seabream in the Ligurian Sea. It found that farmed specimens exhibited significantly lower concentrations of mercury than wild specimens.<br>- The percentage of organic mercury was slightly higher in wild fish (98% vs. 85% in farmed).<br>- No relationship was found between mercury concentration and body size in farmed fish.	-MeHg intake remains below the recommended limits even if the amount of farmed fish consumed is four times higher than wild fish. This may be important for sensitive subjects such as children less than 15-years old.
	Sparus aurata	5	Wild	meat	0.29	0.72	0.54		

**Miniero R. 2014 (33)**	*Auxis rochei Risso*		Adriatic, Ionian, and Tyrrhenian seas	Muscle	0.055	2	0.223	- The high HgTOT findings confirm that the Mediterranean Sea is a geological hot spot for mercury. In farmed fish, MeHg and HgTOT concentrations were all below the Q. values of the corresponding distributions observed for 25 wild species. Correlation lines for the THg and MeHg, highly significant.	- Only predatory fish are at risk of exceeding the existing food regulatory maximum levels, an aspect suggesting that management measures for fish and fishery products should be calibrated on a species- or group-specifi basis.
	Dicentrarchus labrax L		Farmed	Muscle	0.033	0.135	0.058		
	Others 12 different species		Adriatic, Ionian, and Tyrrhenian seas	Muscle					
**Nepusz T. 2009 (45)**								- The contaminated seafoods originate in thirty-two countries with only ESP producing over 10 contaminated seafood products	- Food alerts owing to metal contamination during the study period are mainly due to Hg, Cd, and Pb in seafood.
**Novakov NJ. 2017 (66)**	*canned tuna*	57 (Thailand, Vietnam-indonesia)	supermarkets in the region of Vojvodina, Serbia		0.007	0.64	0.18	- Heavy metal levels varied widely:, mercury. Mercury and lead levels were generally below legislative limits	- Obtained results show safe status regarding mercury in investigated canned fish marketed in Serbia.
	canned sardines	25 (Marocco Croatia Russia)			0.007	0.45	0.13		
**Pawlaczyk A. 2020 (74)**	*Canned fish*	84	Local markets in Poland					- None of the 84 canned fish samples analyzed in Poland exceeded the acceptable mercury limits established by FAO/WHO. Although canned tuna, especially that preserved in oil, showed the highest total mercury concentrations.<br>- Hg does not migrate into packaging liquids (oils, sauces), suggesting a strong bond with fish proteins. Predatory fish contained significantly more mercury than non-predatory fish, and those from the Atlantic Ocean showed higher levels than those from closed seas.	- Fish caught in the Atlantic Ocean presented higher mercury content than the ones from closed seas The tolerable weekly intake of mercury for an individual weighing about 70 kg for none of the samples analyzed was exceeded<br>- These levels were not considered alarming.
**Perugini M. 2009 (31)**	*Nephrops norvegicus*	13			0.29	3.27	0.97	- Only N. norvegicus is benthonic while the other species are demersal or pelagic. It can be assumed that Norway lobster showed the highest levels of total Hg because it is a scavenging animal that feeds on a wide range of decomposition residues, most constituting humic.<br>- The results of this study showed that more than 25% of samples exceeded 0.5 mg/kg of total Hg.	- This estimated value exceeded the above-mentioned TDI (0.23 lg/kg-bw) and it should be higher if considering populations with a high consumption of fishery products. pregnant and breastfeeding women as well as young children select fish from a wide range of species, without giving undue preference to large predatory fish.
	Mullus barbatus	14			0.05	1.07	0.48		
	Todarodes sagittatus	14			0.04	1.99	0.25		
	Scomber scombrus	14			0.06	1.42	0.36		
	Micromesistius poutassou	13			0.03	1.17	0.38		
	Merluccius merluccius	14			0.02	0.62	0.59		
**Polak-Juszczak L. 2012 (62)**	*Platichtys flesus*	74	southern Baltic Sea ICES 26	muscle	0.045	0.11	0.077	- Hg concentrations in dab muscle significantly increased by 1.4% annually. leading to a 41% rise in contamination levels over a 25-year period). a trend found to be independent of fish age.<br>- Hg concentrations in nearby North Sea sediments significantly decreased during the same period. indicating that sediment trends cannot explain the increase observed in fish.<br>The study highlights a contradiction between contamination trends in biota and sediment. emphasizing the importance of simultaneously investigating both environmental compartments for accurate monitoring and risk assessment	- Turbot muscle tissues had the maximum affinity to Hg, which means they best reflect the level of this element in the environment of the Baltic Sea.<br>- The muscle tissues of turbot can be used successfully as biomonitors of Hg content in the Baltic Sea because of their ability to accumulate this metal, their tolerance to metals, the adequate amounts of tissue available for analysis, and the sedentary habits of this species.
	Pleuronectes platessa	51			0.033	0.079	0.05		
	Scophthalmus maximus	50			0.047	0.12	0.11		
	Platichtys flesus		southern Baltic Sea ICES 25		0.033	0.102	0.057		
	Pleuronectes platessa				0.035	0.098	0.04		
	Scophthalmus maximus				0.066	0.141	0.084		
**Polak-Juszczak L. 2023 (84)**	*Belone belone*	40	Baltic sea coast in Puck Bay	muscl e				- THg concentrations in garfish muscles indicated that the European Union limit (0.5 mg/kg ww) was not exceeded in any of the specimens examined	- THg and MeHg concentrations increased with specimen weight and length. The study results indicated that methylmercury risk assessments must be performed on different fish length classes, especially in species that have a wide range of body lengths.
**Raimundo J. 2003 (71)**	*Octopus vulgaris*	59	Portugal					- The abundance of metals in each tissue was consistently . Concentrations in the digestive gland reached one and two orders of magnitude higher than those found in arm and mantle,	- The lack of relations between metal concentration in tissues and biological parameters facilitates the recognition of the observed spatial patterns, suggesting

								corroborating that the digestive gland contains the major storage sites for these elements.<br>- Significantly, metal concentrations in tissues did not vary significantly with size/weight, sex, or sexual stage.<br>- The quantity of Hg was expressed only in d.w.	-The digestive gland functions as a detoxification organ by concentrating elements, particularly which was highly concentrated in this tissue.<br>-The drastic increase of levels in the Northern coast is in line with the contrasting Cd distribution in coastal waters, resulting from higher freshwater
**Rubio C. 2008 (44)**			Gran Canaria, La Palma, Lanzarote and Fuerteventura					- Analyzed 420 samples of regularly consumed food and drink in the Canary Islands, finding that the highest Hg concentrations, ranging from non-detectable to 119 mg were observed in the fish product group.<br>It is important to note that all food groups with regulated Hg content showed levels below the legally set values, and no fish sample exceeded the EU maximum tolerance limit.	- This study reveals that dietary Hg fulfils the PTWI fixed by the FAO/WHO and that there is no risk associated to Hg dietary intake in the Canary Islands.
**Sánchez-Muros MJ. 2018 (29)**	*Mullus surmuletus*	5	Almería Bay, Spain	muscle	0.25	0.39	0.31	- In this study. the Hg content of tissues was connected with length. weight. age. trophic level and muscle composition (protein. lipids. ash. etc.). and showed a positive association with age and trophic level.	- The hake, red mullet, bullet tuna and mackerel caught and eaten from Almería do not represent a health risk.<br>- The target organ for Hg accumulation, liver or muscle, depends on species.<br>- Age and trophic levels are related with Hg levels in muscle, but not with Hg in liver.
	Merluccius merluccius	5			0.55	1.06	0.85		
	Auxis rochei	5			0.41	0.79	0.57		
	Scomber japonicus	5			0.28	0.83	0.49		
**Signa G. 2017 (36)**	*Bothus podas*	5	south of the Augusta harbour (Central Mediterranean Sea)				0.50	- THg concentrations significantly increased along trophic position and from pelagic to benthic sedentary fish, revealing a clear influence of trophic position and habitat use on T-Hg accumulation.<br>- Approximately 70% of the analyzed fish species (14 out of 20) exceeded the Maximum Permissible Limit (MPL) for T-Hg in muscle for human consumption, while 9 out of 20 species exceeded the MPL for Cd.<br>- T-Hg concentrations were positively correlated with fish size for most species, while (TMFs) were 1.22 for THg, indicating biomagnification, and 0.83 for Cd, indicating biodilution across the entire food web.	- Diet and habitat use shaped the mercury burden in fish from Priolo Bay, but not that of cadmium.<br>- Mercury was found to be more bioavailable for benthic fish due to significant sediment contamination in Augusta Bay and photo-demethylation in the water column.<br>- Augusta Harbour is a source of Hg for the adjacent Priolo Bay, providing evidence of consistent trophic transfer from contaminated sediment to biota, up to higher trophic level fish.
	Coris julis juv.	5					2.77		
	Dactylopterus volitans	3					0.97		
	Dasyatis pastinaca juv.	2					1.09		
	Dentex dentex juv.	5					1.51		
	Diplodus annularis	5					1.68		
	Lithognathus mormyrus	5					1.3		
	Liza saliens	3					0.07		
	Mullus surmuletus	3					0.62		
	Muraena helena	5					4.8		
	Pagellus erythrinus	5					0.71		
	Pagrus pagrus juv.	5					1.03		
	Raja radula	2					0.79		
	Sarpa salpa	3					0.14		
	Scorpaena porcus	5					4.4		
	Scorpaena scrofa	4					3.98		
	Serranus scriba	4					5.5		
	Sparisoma cretense	4					0.13		
	Symphodus tinca	4					1.35		
	Xyrichtys novacula	2					1.38		
**Squadrone S. 2013 (37)**	*Mugil cephalus*		Bocca di Magra, La Spezia (Ligurian Sea, Mediterranean Sea, Italy)	Muscle	< 0.1		< 0.1	analyzed the concentrations mercury (Hg) in the muscle tissue of 200 flathead mullet (Mugil cephalus) specimens collected from the Ligurian Sea.<br>- None of the samples exceeded the regulatory limits established by the European Union.<br>- Hg levels were negligible or below the limit of quantification (LOQ).	- La Spezia, Ligurian Sea, had a mercury, content under the legislation limit. In particular, mercury and cadmium levels in muscles of M. cephalus were negligible and consumption of M. cephalus from North-West Mediterranean Sea is not a problem for human health.
**Stamatis N 2019 (55)**	*Thunnus alalunga*	82	Aegean Sea	Muscle	0.14	0.94	0.44	None of the samples contained mercury above the limit	Concentrations of toxic heavy metals in albacore, especially mercury, must be monitored regularly and comprehensively with respect to consumer health.

**Storelli MM. 2000 (16)**	*Lepidorhombus boscii*	112	Adriatic Sea along the Apulian coast	Meat	0.05	0.92	0.39	- Considering a weekly average consumption product of 420 g per capita and the mean total mercury values found in every single species.<br>- All the samples analysed except angler fish presented estimated dietary exposure values lower than that recommended by JEFCA for THg.	- Lophius piscatorius and Lophius budegassa from the South Adriatic Sea exhibited the highest total mercury concentrations in their muscle tissue, with a substantial number of samples exceeding European legal limit.<br>- Other benthic species, such as megrim and striped mullet, also had samples surpassing the limit.<br>- A clear correlation was found between the total mercury concentration and the weight of the fish.<br>- The research emphasized that mercury levels in these benthic species often exceeded regulatory limits and dietary exposure recommendations, posing a potential health risk to human consumers
	Solea vulgaris	100			0.05	0.44	0.19		
	Mullus barbatus	312			0.1	0.63	0.31		
	Lophius piscatorius	120			0.61	2.22	1.26		
	Lophius budegassa	156			0.22	1.62	0.68		
**Storelli MM. 2001 (85)**	*Xiphias gladius*	162	Thyrrenian Sea	Muscle	0.15	1.05	0.49	- The mean mercury concentrations were about two to four times higher in blue in tuna than in swordfish when the weight ranges of fish were the same.<br>- Tunas have rates of digestion two to five times higher than those of other piscivorous species when comparisons are made to other fishes of equal body size held at equivalent water temperatures.<br>- Bluefin tuna from the Mediterranean Sea generally exhibited higher total mercury concentrations in their muscle tissue than swordfish, with a significant proportion of both species, especially bluefin tuna, exceeding the European legal limits.	- A strong positive correlation was observed between mercury accumulation and fish size for both species. The observed differences in mercury levels were attributed to varying feeding behaviors (bluefin tuna being more piscivorous) and physiological growth rates.
	Thunnus thynnus	169	Thyrrenian Sea	Muscle	0.07	4.26	1.02		
**Storelli MM. 2002 (4)**	*Thunnus alalunga*	127	Adriatic sea	muscle	0.84	1.45	1.17	- The study determined THg and MeHg levels in the muscle tissue of albacore (Thunnus alalunga) and bluefin tuna (Thunnus thynnus) caught in the Mediterranean Sea.<br>- Average total mercury concentrations were 1.17 mg/kg wet weight for albacore and 1.18 mg/kg wet weight for bluefin tuna. A high percentage of samples exceeded the legal limit set by the European Commission.	- The consumption of Mediterranean albacore and bluefin tuna may pose a risk to human health due to high mercury concentrations<br>- The significant exceedance of the PTWI demands greater attention.
	Thunnus thynnus	161	Ionian sea	muscle	0.16	2.59	1.18		
**Storelli MM. 2002 (34)**	*Chimaera monstrosa*	160	South Adriatic Sea		1.3	5.16	3.14	- For Gost shark: the levels of Hg in fish increase with body size, so larger, older fish have generally higher concentrations than smaller, younger fish.<br>- Total body length and body weight were significantly correlated	- Therefore, the consistent levels in ghostshark and electric ray may be attributed either to the habitat or their high trophic position.<br>- The results have demonstrated that high levels of Hg, mainly as MeHg, can be accumulated by animals at the top of the food chain and that the accumulation in fish is influenced by quite a number of factors, among which size, fish ecology and feeding habits
	Torpedo nobiliana	27			1.65	3.59	2.42		
	Myliobatis aquila	15			0.67	1.01	0.83		
**Storelli MM. 2003 (46)**	*Raja oxyrhynchus*	13	Southern areas of the AdrIatic and Ionian Seas of Italy	Muscl e			1.67	- Animal size is recognized to be of importance in determining the rate of physiological processes influencing the uptake, distribution, and elimination of pollutants mercury accumulates in organisms mainly as MeHg because of the lipophilic nature of this compound, which facilitates its penetration into the cell	- The results show that in the muscle tissue of all of the species analyzed, mercury was present mainly in the organic form, while inorganic mercury made a negligible contribution to the total mercury burden. - Comparison with the PTWI shows that mercury intake through the consumption of some sh species could constitute cause for concern, because the PTWI was clearly exceeded.
	Raja clavata	31					1.1		
	Raja miraletus	18					0.96		
	Raja asterias	27					0.81		
	Micromesistius potassou	70					0.7		
	Mullus barbatus	154					0.7		
**Storelli MM. 2003 (17)**	*Trachurus trachurus*	100	Central and south adriatic sea	Muscle	N.D.	1.87	0.23	- The form of mercury continuously taken up by the animals through their diet is essentially MeHg.<br>- The methylmercury proportions to total mercury recorded in the present study varied between 70 and 100%.	- Dietary intake depends either on the concentration levels in various fish or on the amounts of foods consumed.<br>- The estimated weekly intake calculated using mean concentrations of THg and MeHg ranged from 0.44 to 5.59 mg/kg1 body weight and from 0.44 to 5.22 mgkg1 body weight, respectively. A higher exposure was associated with the consumption of skates, frost fish and angler fish, while the consumption of megrim, four spotted megrim, red fish, striped mullet and forkbeard gave weekly intakes slightly below the established PTWI.
	Sardinella aurita	150			N.D.	0.3	0.09		
	13 others species	2960							

**Storelli MM. 2004 (24)**	*Thunnus alalunga*	136	Adriatic Sea	Flesh	0.88	2.34	1.56	- Consumption of the flesh of large predatory fish may be of concern for human health	- Albacore flesh is mainly used to prepare canned tuna, whose consumption/day per capita is quite limited, it is likely that the dietary exposure for mercury might be overestimated
	Eledone moschata	310	Adriatic Sea	Liver	0.95	4.3	2.41		
**Storelli MM. 2005 (76)**	*Patella caerulea*		Punta rondinella Ionian Sea		N.D.	0.09	0.05	- The study investigated concentrations of mercury (Hg). […] in bioindicator organisms such as limpets (Patella caerulea) and mullets (Mullus barbatus) in the Ionian Sea.<br>- Elevated mercury concentrations were found in mullet specimens from Sicily. particularly Capo Passero. Mullets from Capo Passero exhibited significantly higher mercury levels compared to other stations. potentially influenced by fish size and the area's hydrodynamics.	- The high mercury levels in samples from Capo Passero station are of great concern with regard to environmental health, needing, thus, a long term monitoring programme.
	Patella caerulea		Lido azzurro Ionian Sea		N.D.	0.09	0.05		
	Mullus barbatus		Roccella jonica		N.D.	0.3	0.12		
	Mullus barbatus		Catania		Trace	0.25	0.11		
	Mullus barbatus		Capo passero		0.31	1.5	0.81		
**Storelli MM. 2013 (18)**	*Conger conger*	149	Adriatic Sea (Mediterranean Sea)		0.57	1.98	1.14	- Hg was the most abundant element in the species studied, followed by Pb and Cd, which exhibited comparable levels.	- EWI the higher estimated values, accounting from 33.8% to 54.6% of PTWI of total Hg, were associated to consumption of larger size fish of the examined species.<br>- THQ, being higher than critical value of 1, suggest that these species, especially those of larger size, must be eaten in moderation.
	Raja miraletus	127			0.4	1.78	1.09		
	Lophius budegassa	105			0.23	1.76	0.96		
	Helicolenus dactylopterus	243			0.24	1.9	1.04		
	Mullus barbatus	287			0.05	1.85	0.43		
**Visciano P. 2015 (58)**	*Chamelea gallina*	2250	Abruzzi coastal region of the central Adriatic Sea	body of clams	< 0.0050			- The central Adriatic Sea is less affected by pollution effluents than is the northern sea, where the Po River drains a very industrialized and intensively cultivated area	- The results revealed that C. gallina from the central Adriatic Sea could be considered safe about the contaminants studied
**Wilman B. 2019 (64)**	*Rhithropanopeus harrisii*	92	Southern Baltic Sea					- HgT concentration increased with the carapace size (age) of specimens.	- The accessibility of R. harrisii as a food due to its widespread distribution and abundance will contribute to the introduction of smaller load of toxic mercury into the Puck Bay food chain, at the end of which is a human.
